# Long non-coding RNA UCA1 promotes malignant phenotypes of renal cancer cells by modulating the miR-182-5p/DLL4 axis as a ceRNA

**DOI:** 10.1186/s12943-020-1132-x

**Published:** 2020-01-29

**Authors:** Wei Wang, Wentao Hu, Ya Wang, Yong An, Lei Song, Panfeng Shang, Zhongjin Yue

**Affiliations:** 1 Department of Urology, Institute of Urology, Gansu Nephro-Urological Clinical Center, Key Laboratory of Urological Diseases in Gansu Province, Lanzhou University Second Hospital, Lanzhou, 730030 Gansu China; 2 School of Radiation Medicine and Protection, Medical College of Soochow University, Collaborative Innovation Center of Radiological Medicine of Jiangsu Higher Education Institutions, Suzhou, 215123 China; 3 Department of Nephrology, Second Hospital Lanzhou University Second Hospital, Lanzhou, 730000 Gansu China; 4 Medical School, Northwest Min Zu University, Lanzhou, 730030 Gansu China

**Keywords:** UCA1, miR-182-5p, ceRNA, DLL4, Renal cancer

## Abstract

**Background:**

Accumulating literatures have indicated that long non-coding RNAs (lncRNAs) are potential biomarkers that play key roles in tumor development and progression. Urothelial cancer associated 1 (UCA1) is a novel lncRNA that acts as a potential biomarker and is involved in the development of cancers. However, the molecular mechanism of UCA1 in renal cancer is still needed to further explore.

**Methods:**

The relative expression level of UCA1 was determined by Real-Time qPCR in a total of 88 patients with urothelial renal cancer and in different renal cancer cell lines. Loss-of-function experiments were performed to investigate the biological roles of UCA1 and miR-182-5p on renal cancer cell proliferation, migration, apoptosis and tumorigenicity. Comprehensive transcriptional analysis, dual-luciferase reporter assay and western blot etc. were performed to explore the molecular mechanisms underlying the functions of UCA1.

**Results:**

In this study, we found that UCA1 was significantly up-regulated in renal cancer. Moreover, increased UCA1 expression was positively correlated with differentiation and advanced TNM stage. Further experiments demonstrated that knockdown of UCA1 inhibited malignant phenotypes and Notch signal path of renal cancer cells, and miR-182-5p was reverse function as UCA1. UCA1 functioned as a miRNA sponge to positively regulate the expression of Delta-like ligand 4(DLL4) through sponging miR-182-5p and subsequently promoted malignant phenotypes of renal cancer cells, thus UCA1 playing an oncogenic role and miR-182-5p as an antioncogenic one in renal cancer pathogenesis.

**Conclusion:**

UCA1-miR-182-5p-DLL4 axis is involved in proliferation and progression of renal cancer. Thus, this study demonstrated that UCA1 plays a critical regulatory role in renal cancer cell and UCA1 may serve as a potential diagnostic biomarker and therapeutic target of renal cancer.

**Supplementary Information:**

The online version contains supplementary material available at 10.1186/s12943-020-1132-x.

## Introduction

Renal cell carcinoma (RCC) accounts for approximately 3% of all adult cancers, and poor survival is manifested in RCC patients, especially for those with metastasis [1]. RCC exerts socio-economic burden on society and the families of patients. However, the pathogenesis of RCC remains unclarified.

Various literatures reported that cancers are closely related to the abnormal expression of LNCRNA, miRNA and proteins etc., and even co-functions each other. With the rapid development of sequencing technologies, a series of dysregulated long non-coding RNAs (lncRNAs) have been found in many human diseases, especially in cancers [2–8]. Accumulating evidences suggested that long non-coding RNAs (lncRNAs) perform important or vital functions in the malignant tumors [9–15]. LncRNA urothelial cancer associated 1 (UCA1) is abnormally expressed in esophageal squamous cell carcinoma, colorectal cancer, gastric cancer melanoma cells, pancreatic cancer, thyroid cancer, lung cancer and so on [16–30]. In vitro and in vivo assays were conducted to further explore its underlying roles in tumor progression. In this study, we illustrated that UCA1 promoted tumor cell proliferation or migration and suppressed apoptosis of renal cancer and the mechanism of action.

MicroRNAs (miRNAs) are non-coding RNAs with 18–25 nucleotides, which participate in various processes of tumorigenesis. The expression of miR-182-5p is not consistent across tumors. MiR-182-5p is down-regulated in ovarian cancer, colorectal cancer and renal cancer etc. However, miR-182-5p is up-regulated in Oral squamous cell carcinoma, breast cancer and hepatocellular carcinoma etc. [31–36]. However, the underlying mechanisms of miR-182-5p in the malignant behaviors of RCC are unclarified. In this study, we demonstrated that miR-182-5p as negative regulatory factor were involved in the progression of renal cancer as well.

Accumulated evidences reported lncRNAs function as miRNAs sponges. This is firstly reported the relation between UCA1 and miR-182-5p as miRNAs sponges in the renal cancers in the world. This study helps broaden our knowledge of the expression pattern.

Additionally, proteins are the necessary substance to participate in lives’ activities and disease progression as well, which were closely related with lncRNA and miRNA etc. Delta-like ligand 4 (DLL4), one of the ligands of Notch receptors, is predominantly expressed in the endothelial cells and has been shown to play a pivotal role in regulating tumor angiogenesis. During angiogenesis, activation of the Notch/DLL4 pathway selects the stalk cell, and promotes endothelial basement membrane formation and cell adhesion. Some studies reveal a role for DLL4 in tumorigenesis in several cancers, including T acute lymphoblastic leukemia (T-ALL), and glioblastoma etc. [37–47]. DLL4 is one of the ligands of Notch 1. Activation of DLL4-NOTCH signaling is important for the RCC to maintain its malignant properties. DLL4 overexpression significantly reversed cell proliferation inhibition and migration of renal cancer cells induced by silencing UCA1, and remarkably reversed cell apoptosis promotion of renal cancer cells induced by silencing UCA1. DLL4-NOTCH signaling is critical for the progression of renal cancer cells.

In the present study, we showed that UCA1 was involved in the progression of renal cancer. Mechanistically, we found that UCA1 functioned as a miRNA sponge to positively regulate Delta-like ligand 4(DLL4) expression by sponging miR-182-5p in a ceRNA-dependent manner. Together, our results suggest that UCA1 is a powerful tumor biomarker, and UCA1-miR-182-5p-DLL4 axis is involved in proliferation, migration, apoptosis and progression of renal cancer, which highlight its potential clinical utility as a promising diagnostic and therapeutic target of renal cancer. Our findings may provide a new horizon for exploring therapeutic target of renal cancer.

## Materials and methods

### Patient samples

Eighty eight renal cancer patients (Renal clear cell carcinoma) and 30 renal cancer patients (Papillary renal carcinoma) who received radical nephrectomy were included in this research. Renal cancer tissues and matched normal peritumoral tissues were snap-frozen in liquid nitrogen quickly after resection. Written informed consent was also obtained from all the patients. The study was approved by the institutional research ethics committee of The Second Hospital of Lanzhou University (Granted 2017A-021).

### Cell lines and cell culture

Human renal cancer 786-O, Caki-1, human normal renal epithelial cells (293 T) and RPTEC (Renal Tubular Epithelial Cell) were purchased from the Institute of Cell Biology, Chinese Academy of Sciences (Shanghai, China). The 786-O cells were cultured in RPMI-1640 (1640) (HyClone, USA). The Caki-1 cell lines were cultured in McCoy’s 5A (HyClone, USA). The 293 T cells were cultured in DMEM (HyClone, USA). The RPTEC cells were cultured in F12 (HyClone, USA).1640, DMEM, F12 and McCoy’s 5A were mixed with 1% antibiotics (100 U/ml penicillin and 100 μg /ml streptomycin sulfates) and 10% fetal bovine serum (FBS). Then plates were placed in incubator at 37 °C with an atmosphere of 5% CO_2_.

### Cell transfection

Short hairpin RNAs (shRNAs) against UCA1 gene or DLL4 gene were ligated into pGPU6/GFP/Neo vectors (shUCA1, shDLL4) (Gene Pharma, Shanghai, China) and plasmid with non-targeting sequence was used as a negative control (shNC). The microRNA mimics (agomir) and microRNA inhibitor (antagomir) were purchased from Gene Pharma, Shanghai, China. Before transfection, the cells were cultured 24 h. Then, the cells were transiently transfected with corresponding vector using Lipofectamine 3000 Transfection Reagent (Invitrogen, Carlsbad, CA, USA) according to the manufacturer’s instructions. After 48 h, cells transfected with corresponding vector were harvested for quantitative real-time PCR and so on. Experiments were repeated at least three times.

### Real-time quantitative PCR

Total RNA was extracted from the tissues or the transfected cells using TRIzol reagent (Invitrogen, Grand Island, NY, USA) according to the manufacturer’s instructions. The cDNA was synthesized from total RNA(1000 ng) using the Prime Script RT Reagent Kit with gDNA Eraser (Takara, Kyoto, Japan). The expression levels of UCA1 etc. (about 50 ng cDNA, see the relevant specifications for details) were measured by real-time quantitative PCR utilizing SYBR Premix Ex Taq II (Takara) on the CFX96 sequence detection system (Bio-Rad). The primer sequences were as follows in Additional file 1: Table S1. GAPDH and U6 small nuclear RNA were chosen as the internal control. Expressions were normalized to endogenous controls and calculated using relative quantification method (2^-ΔΔCt^). Experiments were repeated at least three times.

Glyceraldehyde 3-phosphate dehydrogenase (GAPDH) or U6was chosen as the endogenous control to normalize the data.

### LncRNAs immunoprecipitation

Biotin-labeled lncRNA-UCA1 probe was synthesized by RiboBio. Renal cancer cells were fixed by 1% formaldehyde for 10 min, lysed and sonicated. After centrifugation, 50 μl of the supernatant was retained as input and the remaining part was incubated with lncRNA-UCA1 specific probes-streptavidin dynabeads (M-280, Invitrogen) mixture over night at 30 °C. Next day, M-280 dynabeads-probes-lncRNAs mixture was washed and incubated with 200 μl lysis buffer and proteinase K to reverse the formaldehyde crosslinking. Finally, the mixture was added with TRIzol for RNA extraction and-qPCR detection. Renal cancer cells were lysed with radioimmunoprecipitation assay (RIPA) buffer containing protease inhibitors (Sigma).

### Cell proliferation assays

Cell Counting Kit-8, CCK-8 (Beyotime Institute of Biotechnology, Shanghai, China) was used for cell proliferation according to the manufacturer’s instructions. For CCK-8 assay, cells were incubated in a 96-well plate for 24 h, and then transiently transfected with siRNAs or plasmids. The absorbance in each well was measured at 0, 24, 48 and 72 h after transfection by a microplate reader (Bio-Rad, Hercules, CA, USA). Experiments were repeated at least three times.

### Ethynyl-2-deoxyuridine (EdU) incorporation assay

Cell proliferation was also determined by Ethynyl-2-deoxyuridine incorporation assay using an EdU Apollo DNA in vitro kit (RIBOBIO, Guangzhou, China) following the manufacturer’s instructions. Briefly, after transfected with corresponding vector cells were incubated with 100 μl of 50 μM EdU per well for 2 h at 37 °C, respectively. Finally, the cells were visualized under a fluorescence microscopy. Experiments were repeated at least three times.

### Cell migration assay

Cell migration was detected by scratch assay according to the reported methods [22]. Cells were seeded in 6-well plates and incubated in incubator to get 100% confluence before transfection. The cells were transfected with siRNAs or plasmids. A sterile 200 μl pipette tip was used to generate a clear line in the wells. Pictures were taken from each well quickly using a digital camera system. After one day, pictures were taken again. Migration distance was counted at the time of 0 h and 24 h. Experiments were repeated at least three times.

### Flow cytometry assay

Renal cancer 786-O and Caki-1 cells were transiently transfected with siRNAs or plasmid vectors. 48 h after transfection, cells were harvested and resuspended in fixation fluid 5 μl Annexin V-FIFC and 100 μl propidium iodide were added to 500 μl cell suspension. Cell apoptosis was then determined by using flow cytometry (EPICS, XL-4, Beckman, CA, USA). In the graphs, the quadrant respectively stands for dead cells, living cells, early apoptotic cells and late apoptotic cells. Experiments were repeated at least three times.

### Western blot analysis

The transfected cells were washed with PBS, and total proteins were extracted using RIPA buffer reagent (Thermo Fisher Scientific). The concentrationofthe lysate was detected by a BCA kit (Thermo Fisher Scientific) following the manufacturer’s protocol. Twenty micrograms(ug) of proteins were separated utilizing 10% SDS-PAGE gels and then transferred into PVDF membranes. The lysates of tissues or cells with equal weight were separated by SDS-polyacrylamide gel electrophoresis (SDS-PAGE) and transferred to PVDF membranes. After blocking in the 5% milk without fat, the membranes were incubated in the primary antibodies for 16 h in 4 °C and then incubated with secondary antibodies at room temperature for 2 h. Immunoblots were visualized using ECL chemiluminescent detection system and analyzed with Chem Imager 5500 V2.03 software. The integrated density values (IDVs) were calculated using Fluor Chem 2.0 software with β-actin as the internal standard. The following antibodies were used as followed as in Additional file 1: Table S2. Goat anti-rabbit antibody and Goat anti-mouse antibody (1:1000, Beyotime, Shanghai, China), Experiments were repeated at least three times.

### Luciferase reporter assay

The predicted binding sites of miR-182-5p with UCA1–3’UTR and DLL4–3’UTR were obtained from RNAhybrid. The binding and mutant sequences were respectively cloned into pmirGLO Dual-luciferase vectors (Gene Pharma, Shanghai, China).786-O, Caki-1 and 293 T cells were cultured in 96-well plates in advance and co-transfected with the wild-type pmirGLO-UAC1 reporter plasmid or the mutated type and mimics-182-5p or NC with Lipofectamine 3000. Cells were also co-transfected with the DLL4–3’UTR reporter plasmid or the mutated type and the above listed mimics. Dual-Luciferase Reporter Assay System (Promega, Madison, WI USA) was used to analyze luciferase activity, which was recorded as the ratio of firefly luciferase activity to renilla luciferase activity. Experiments were repeated at least three times.

### Animal experiments

Institutional Ethics Review Board approved this experimental procedure (Granted D2017–008). A total of 10 male immune-deficient BALB/c nude mice (5–6 wk. old) were purchased from Shanghai lingchang Laboratory Animals (Shanghai, China), and each group was 5 mice. The shUCA1 and shNC vectors were packed into lentivirus (LV-UCA1 and LV-NC) that were purchased from Gene Pharma (Gene Pharma, Shanghai, China). A total of 5 × 10^5–6^ treated Caki-1 cells were suspended in 100 ml Matrigel (BD Biosciences) and injected subcutaneously into the dorsal flank regions of BALB/c nude mice. Tumor growth of mice was monitored every 5 days, and mice were sacrificed 6 weeks, after inoculation. Tumor volume was calculated using the formula, 0.5ab^2^, where “a” and “b” meant long diameter and short diameter, respectively. Finally, mice were executed, and the subcutaneous weight of each tumor was observed. All animal experiments were approved by the Committee of Animal Experimental Ethics. Experiments were repeated at least three times.

### Immunofluorescence

Immunostaining was performed on the paraffin-embedded tumor tissues from nude mice. The avidin-biotin-peroxidase method was adopted to determine the location and relative expression level of the target proteins. The primary antibodies of DLL4, was used at a dilution of 1:2000. Sections were visualized under a fluorescence microscopy (Olympus, Japan). Experiments were repeated at least three times.

### Statistical analysis

All experimental assays were performed in triplicate. All data were presented as mean ± standard deviation (SD) of triplicate biological replicates or samples. All statistical analyses were executed by using SPSS 20.0 software (IBM, Chicago, IL, USA) etc. The UCA1 RNA and miR-182-5p expression differences between renal cancer tissues and matched normal tissues were analyzed using paired samples t-test. CCK-8 assay data were analyzed by ANOVA. The independent samples t-test was used to analyze other data. *P* value of less than 0.05 was considered to be statistically significant.

## Results

### Up-regulation of UCA1 and low-expression of miR-182-5p in renal cancer tissues, cells and both correlation with clinical pathologic factors

**Fig. 1:**
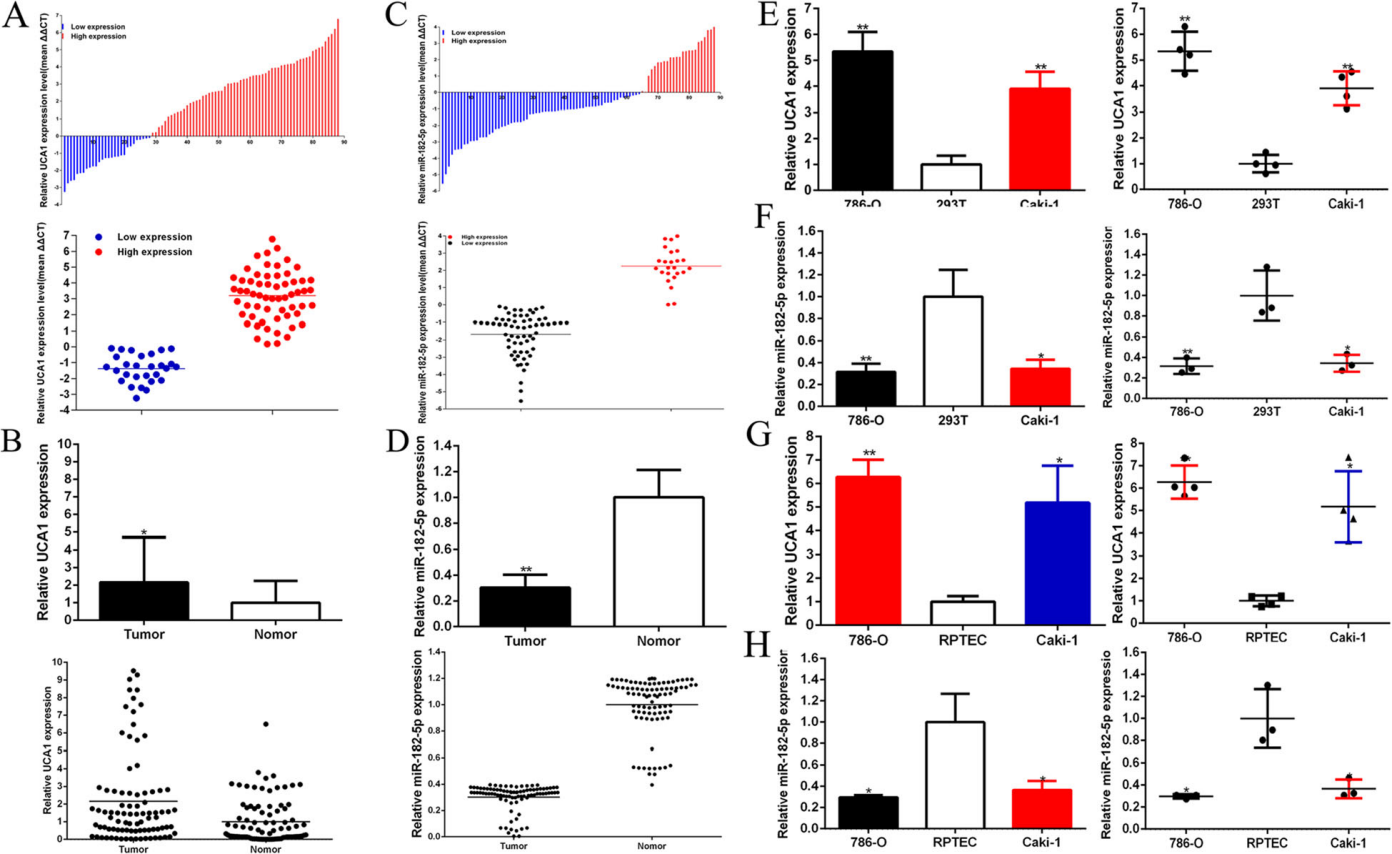
The expression of UCA1and miR-182-5p in renal cancer. Eighty eight patients’ samples were included in the research. GAPDH was the internal control gene in qPCR, which detected relative expression of UCA1 and miR-182-5p in renal cancer tissues and cells. The relative expression patterns of UCA1 (**a** and **b**) and miR-182-5p (**c** and **d**) in paired renal cancer tissues and normal tissues were shown. We also compared its expression level in renal cancer cells (786-Oand Caki-1) and 293 T (**e** and **f**). Assays were performed in triplicate, and data were shown as mean ± standard deviation (SD) of those biological replicates or samples (*P < 0.05, **P < 0.01)

The relative expression level of UCA1 and miR-182-5p was detected by using Real-Time qPCR in a total of 88 patients with renal cancer. Compared to matched normal peritumoral tissues, the UCA1 expression was up-regulated remarkably in 68.2% (60 of 88) of cancer tissues (*P* = 0.021), and about 2.12 times (Fig. 1a and b),and the miR-182-5p expression was down-regulated remarkably in 72.7% (64 of 88) of cancer tissues (*P* = 0.002) (Fig. 1c and d). Compared with the 293 T, the UCA1 expression was increased remarkably in both renal cancer cells, 786-O (*P* < 0.001) and Caki-1 (*P* < 0.001) (Fig. 1e),and the miR-182-5p expression was significantly decrease in both renal cancer cells, 786-O (*P* < 0.010) and Caki-1 (*P* = 0.012) (Fig. 1f). Compared with the RPTEC, the UCA1 expression was increased remarkably in both renal cancer cells, 786-O (*P* < 0.001) and Caki-1 (*P* = 0.002) (Fig. 1g),and the miR-182-5p expression was significantly decrease in both renal cancer cells, 786-O (*P* = 0.010) and Caki-1 (*P* = 0.017) (Fig. 1h). As shown in Tables 1 and 2, up-regulated UCA1 was positively associated with renal cancer differentiation (*P* = 0.010), and down-regulated miR-182-5p was positively associated with renal cancer differentiation (*P* = 0.008) and TNM stage (*P* = 0.012). But gender, age, tumor size, and lymph node metastasis had no relation with UCA1 and miR-182-5p expression level. These results indicated that long non-coding UCA1 should play oncogenic role and miR-182-5p should play antioncogene one in renal cancer.

**Table 1:** Correlation between UCA1 expression and clinicopathological characteristics of renal cell cancer patients [3, 48, 49] (Clear cell renal cell carcinoma)

Characteristics	Total	Expression of UCA1		*P* value
		High (*n* = 60)	Low (*n* = 28)	
Gender				
Male	47	35 (74.5%)	12 (25.5%)	0.251
Female	41	25 (61.0%)	16 (39.0%)	
Tumor size (cm)				
≤ 7 cm	50	33 (66.0%)	17 (34.0%)	0.651
>7 cm	38	27 (71.0%)	11 (28.9%)	
Age				
≤ 55	42	28 (66.7%)	14 (33.3%)	0.821
> 55	46	32 (69.6%)	14 (30.4%)	
Differentiation				
Moderate/poor	50	40 (68.2%)	10 (31.8%)	0.010*
Well	38	20 (52.6%)	18((47.4%)	
TNM stage				
T0–1	26	14 (53.8%)	12 (46.2%)	0.080
T2–4	62	46 (74.2%)	16 (25.8%)	
Lymph node metastasis(N)				
N0	79	56 (70.9%)	23 (29.1%)	0.136
N1 or above	9	4 (44.4%)	5 (55.6%)	

(*P < 0.05, **P < 0.01)

TNM according to staging TNM of American Joint Committee on Cancer (AJCC) in 2010

**Table 2:** Correlation between miR-182-5p expression and clinicopathological characteristics of renal cell cancer patients [3, 48, 49] (Clear cell renal cell carcinoma)

Characteristics	Total	Expression of miR-182-5p		*P* value
		High (*n* = 24)	Low (*n* = 64)	
Gender				
Male	47	11 (23.4%)	36 (76.6%)	0.474
Female	41	13 (31.7)	28 (68.3%)	
Tumor size (cm)				
≤ 7 cm	50	16 (32.0%)	34 (68.0%)	0.335
>7 cm	38	8 (21.1%)	30 (78.9%)	
Age				
≤ 55	43	15 (34.9%)	28 (65.1%)	0.152
> 55	45	9 (20.0%)	36 (80.0%)	
Differentiation				
Moderate/poor	50	8 (16.0%)	42 (84.0%)	0.008**
Well	38	16 (42.1%)	22((57.9%)	
TNM stage				
T0–1	26	12 (11.5%)	14 (88.5%)	0.017*
T2–4	62	12 (38.7%)	50 (61.3%)	
Lymph node metastasis(N)				
N0	79	21 (26.6%)	58 (73.4%)	0.700
N1 or above	9	3 (33.3%)	6 (66.7%)	

(*P < 0.05, **P < 0.01)

TNM according to staging TNM of American Joint Committee on Cancer (AJCC) in 2010

### Knockdown of UCA1 and up-regulation of miR-182-5p inhibited cell proliferation of renal cell lines. Up-regulation of UCA1 and down-regulation of mi-182-5p promoted cell proliferation of renal cell lines

**Fig. 2:**
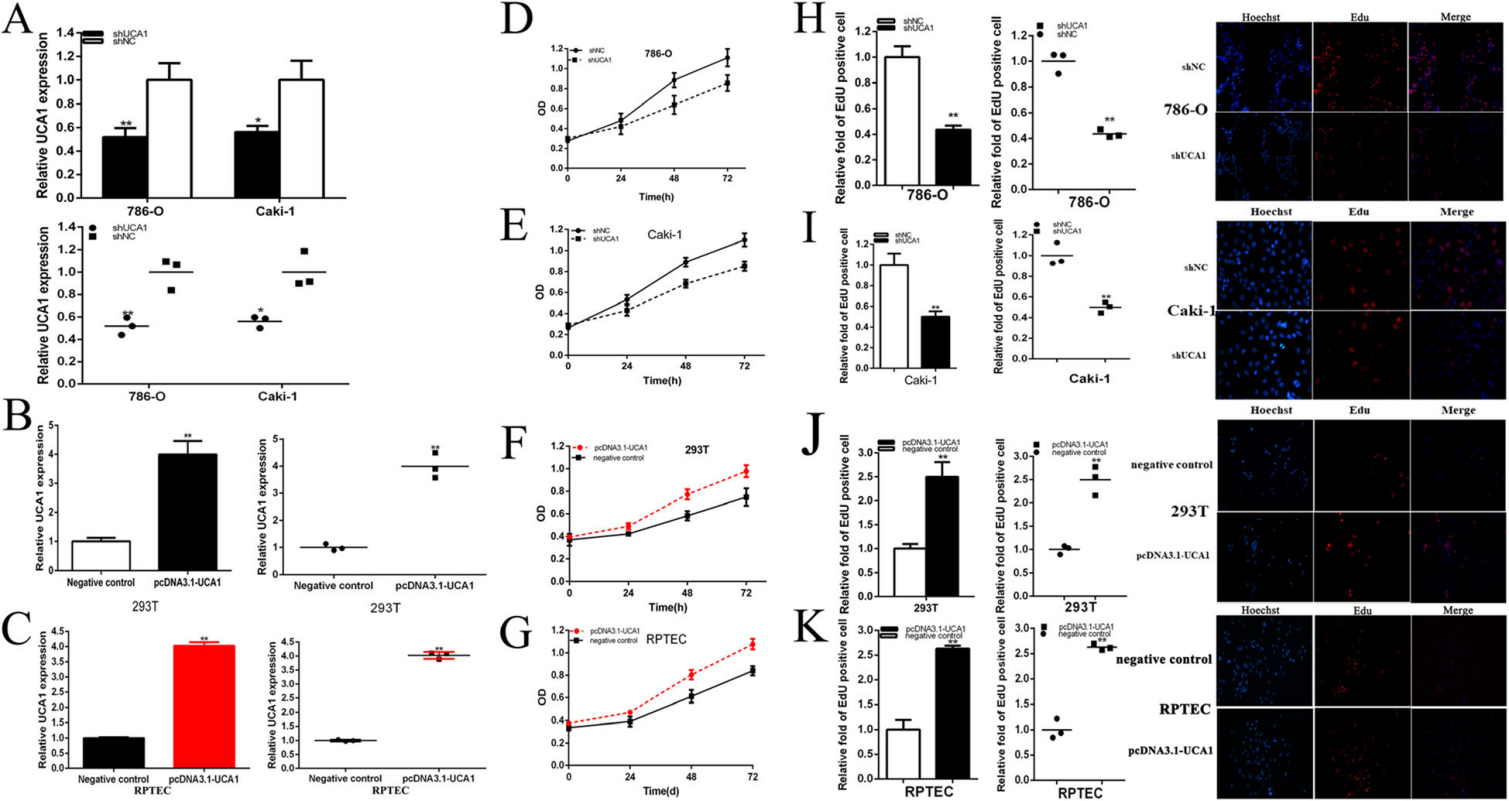
Knockdown and overexpression of UCA1 inhibited or promote cell proliferation. The relative expression level of UCA1 was significantly down-regulated by shUCA1 (**a**) and upregulated by pcDNA3.1-UCA1(**b** and **c**). ANOVA was used for the comparison of curves of cell proliferation. Cell proliferation was detected in both renal cancer cells after transfection of shRNA (**d** and **e**) and pcDNA3.1-UCA1 (**f** and **g**). Representative images of EdU assay and the relative fold changes of EdU positive cells were detected by shRNA (H and I) and pcDNA3.1-UCA1 (**j** and **k**). Assays were performed in triplicate, and data were shown as mean ± standard deviation (SD) of those biological replicates or samples (*P < 0.05, **P < 0.01)

**Fig. 3:**
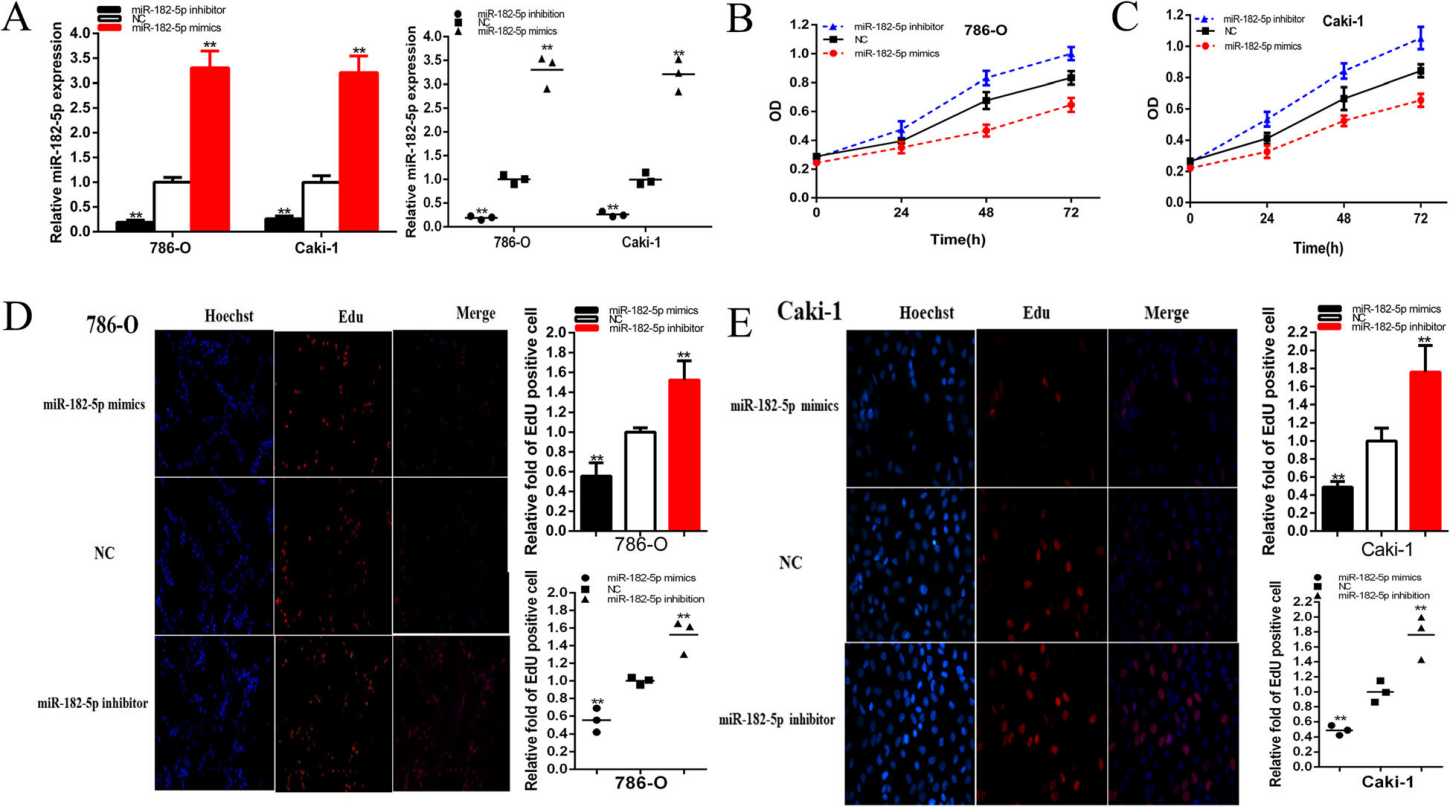
Knockdown and overexpression of miR-182-5p promote or inhibited cell proliferation. The relative expression level of miR-182-5p was significantly down-regulated by miR-182-5p inhibitor and up-regulated by miR-182-5p mimics (**a**). ANOVA was used for the comparison of curves of cell proliferation. Cell proliferation was detected in both renal cancer cells after transfection of miR-182-5p inhibitor and miR-182-5p mimics (**b** and **c**). Representative images of EdU assay and the relative fold changes of EdU positive cells were detected by miR-182-5p inhibitor and miR-182-5p mimics (**d** and **e**). Assays were performed in triplicate, and data were shown as mean ± standard deviation (SD) of those biological replicates or samples (*P < 0.05, **P < 0.01)

We further determined whether UCA1 promotes cell proliferation and miR-182-5p restrained cell proliferation in renal cancer. The relative expression level of UCA1 and miR-182-5p were analyzed by qRT-PCR at 48 h after transfection of shRNA, miRNA mimics or inhibitor in in 786-O and Caki-1 cell lines, and after transfection of pcDNA3.1-UCA1 in 293 T and RPTEC cell line. The relative expression levels of UCA1 was decreased by 48.17% in 786-O (*P* = 0.007) and was decreased by 43.84% in Caki-1(*P* = 0.011) cells were down-regulated significantly by shUCA1 at 48 h post transfection (Fig. 2a). And the relative expression levels of UCA1 was up-regulated significantly in by 3.99 times in 293 T cells (*P* < 0.001) at 48 h post transfection of pcDNA3.1-UCA1 (Fig. 2b). And the relative expression levels of UCA1 was up-regulated significantly in by 4.026 times in RPTEC cells (*P* < 0.001) at 48 h post transfection of pcDNA3.1-UCA1 (Fig. 2 c). And the relative expression levels of miR-182-5p were down-regulated significantly by 80.74% in 786-O (*P* < 0.001) and by 73.75% in Caki-1(*P* < 0.001) cells at 48 h post transfection of miR-182-5p inhibitor (Fig. 3a). And the relative expression levels of miR-182-5p were up-regulated significantly in by 2.30 times in 786-O (*P* < 0.001) and 2.21 times in Caki-1(*P* < 0.001) cells at 48 h post transfection of miR-182-5p mimics (Fig. 3a).

CCK-8 assay was carried out to detect whether shUCA1 or miR-182-5p mimics inhibited the proliferation and pcDNA3.1-UCA1 or miR-182-5p inhibitor promoted the proliferation in 786-O, Caki-1,293 T and RPTEC renal cancer cells. The results demonstrated that shUCA1(Fig. 2d and e) and miR-182-5p mimics (Fig. 3b and c) inhibited cell proliferation remarkably in both renal cancer cells (*P* < 0.01 in two cell lines). PcDNA3.1-UCA1 in 293 T and RPTEC cell lines (Fig. 2f and g), and miR-182-5p inhibitor in both renal cancer cells (Fig. 3b and c) promoted cell proliferation remarkably (*P* < 0.01 in cell lines).

EdU assay was further illustrated to detect cell proliferation. As shown in Fig. 2h-k and Fig. 3d-e, compared to shNC, NC or negative control group, EdU positive 786-O and Caki-1 cells in shUCA1 or miR-182-5p mimics groups were reduced and pcDNA3.1-UCA1 or miR-182-5p inhibition groups were reverse after transfection.

EdU assay illustrated that the quantity of EdU positive cells in shUCA1 group was reduced by 56.37% in 786-O (*P* < 0.001) (Fig. 2h) and decreased by 49.94% in Caki-1 (*P* = 0.002) (Fig. 2i). The quantity of EdU positive cells in pcDNA3.1-UCA1 group was elevated by 2.50 times in 293 T (*P* = 0.001) (Fig. 2j). The quantity of EdU positive cells in pcDNA3.1-UCA1 group was elevated by 2.629 times in RPTEC (*P* < 0.001) (Fig. 2j).

The quantity of EdU positive cells in miR-182-5p mimics group was reduced by 44.47% in 786-O (*P* = 0.005) and decreased by 51.17% in Caki-1 (*P* = 0.005) (Fig. 3d and e). The quantity of EdU positive cells in miR-182-5p inhibitor group was elevated by 1.52 times in 786-O (*P* = 0.010) and raised by1.76 times in Caki-1 (*P* = 0.016) (Fig. 3d and e).

These results demonstrated that knockdown of UCA1 and elevation of miR-182-5p inhibited cell proliferation in renal cell lines and up-regulation of UCA1 and inhibition of miR-182-5p promoted cell proliferation of renal cell lines.

### Knockdown of UCA1 and up-regulation of miR-182-5p inhibited cell migration of renal cell lines. Up-regulation of UCA1 and down-regulation of mi-182-5p promoted cell migration of renal cell lines

**Fig. 4:**
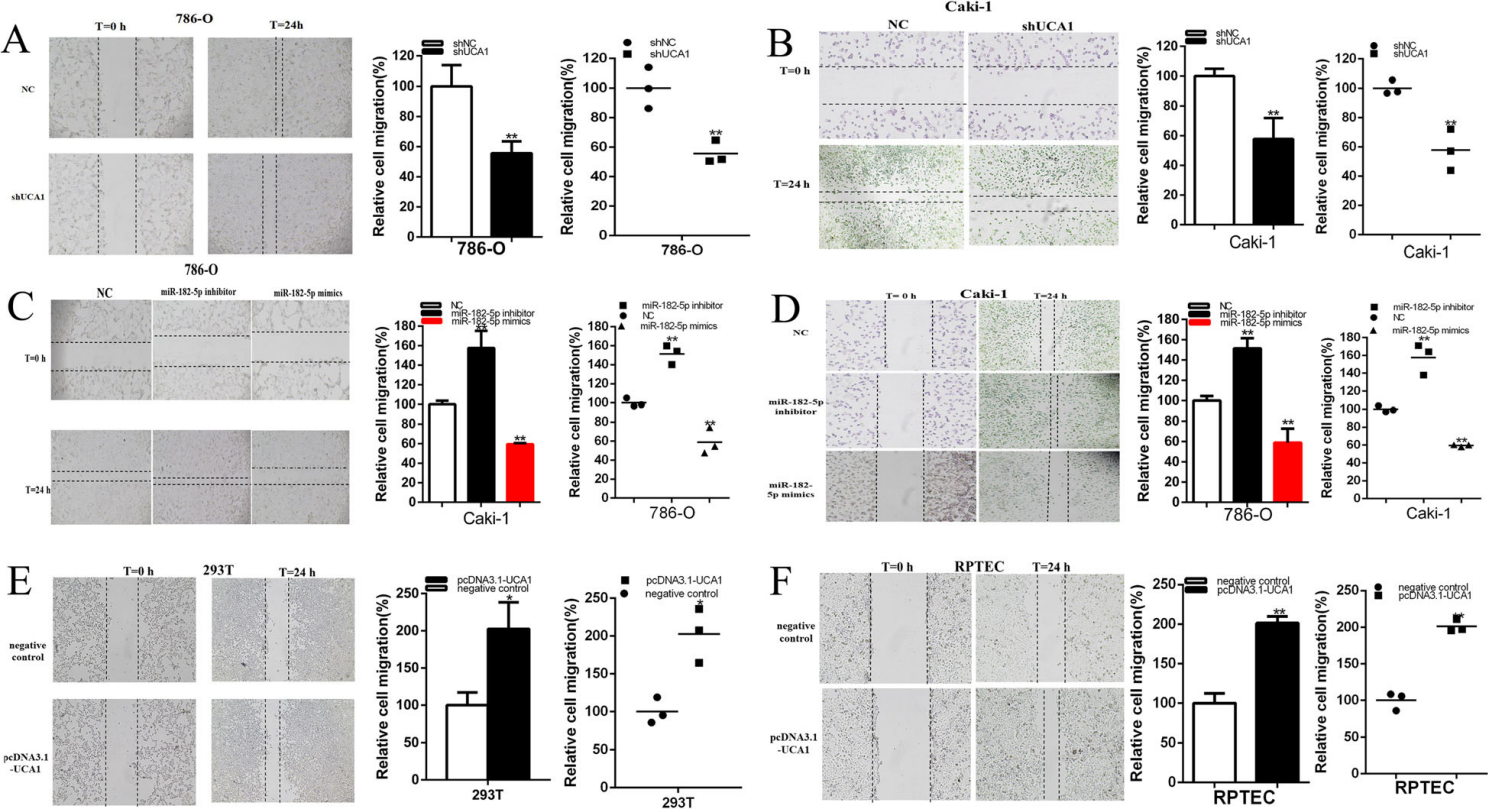
UCA1 and miR-182-5p impacted the renal cancer cell migration. The relative cell migration was suppressed after transfection of shRNA in the 786-O and Caki-1(**a** and **b**) cell lines. The relative cell migration was suppressed or promoted after transfection of miR-182-5p mimics or inhibitor in the 786-O and Caki-1(**c** and **d**) cell lines. The relative cell migration was promoted after transfection of pcDNA3.1-UCA1 in the 293 T and RPTEC cell lines (**e** and **f**). Assays were performed in triplicate, and data were shown as mean ± standard deviation (SD) of those biological replicates or samples (*P < 0.05, **P < 0.01)

Cells were transfected with plasmids, mimics or inhibitor in 6-well plates. The cell scratch assay was utilized to detect the role of plasmids, mimics or inhibitor in cell migration. Compared with shNC group, cell migration of renal cancer cells was significantly suppressed by shUCA1 and miR-182-5p mimics. Scratch assay illustrated that the ratio of the relative migration in shUCA1 group was reduced by 44.35% in 786-O (*P* = 0.009) (Fig. 4a) and decreased by 42.23% in Caki-1 (*P* = 0.008) (Fig. 4b).

The ratio of the relative migration in miR-182-5p mimics group was reduced by 41.12% in 786-O (*P* = 0.008) and decreased by 40.6% in Caki-1 (*P* < 0.001) (Fig. 4c and d). The ratio of the relative migration in miR-182-5p inhibitor group was elevated by 1.51 times in 786-O (*P* = 0.001) and raised by1.58 times in Caki-1 (*P* = 0.005) (Fig. 4c and d). The ratio of the relative migration in pcDNA3.1-UCA1 group was increased by 2.02 times in 293 T (*P* = 0.011) (Fig. 4e). The ratio of the relative migration in pcDNA3.1-UCA1 group was increased by 2.014 times in RPTEC (*P* < 0.001) (Fig. 4f).

These data suggested that knockdown of UCA1 and up-regulation of miR-182-5p inhibited cell migration of renal cell lines. Up-regulation of UCA1 and down-regulation of mi-182-5p promoted cell migration of renal cell lines.

### Knockdown of UCA1 and up-regulation of miR-182-5p promoted cell apoptosis of renal cell lines. Up-regulation of UCA1 and down-regulation of mi-182-5p suppressed cell apoptosis of renal cell lines

**Fig. 5:**
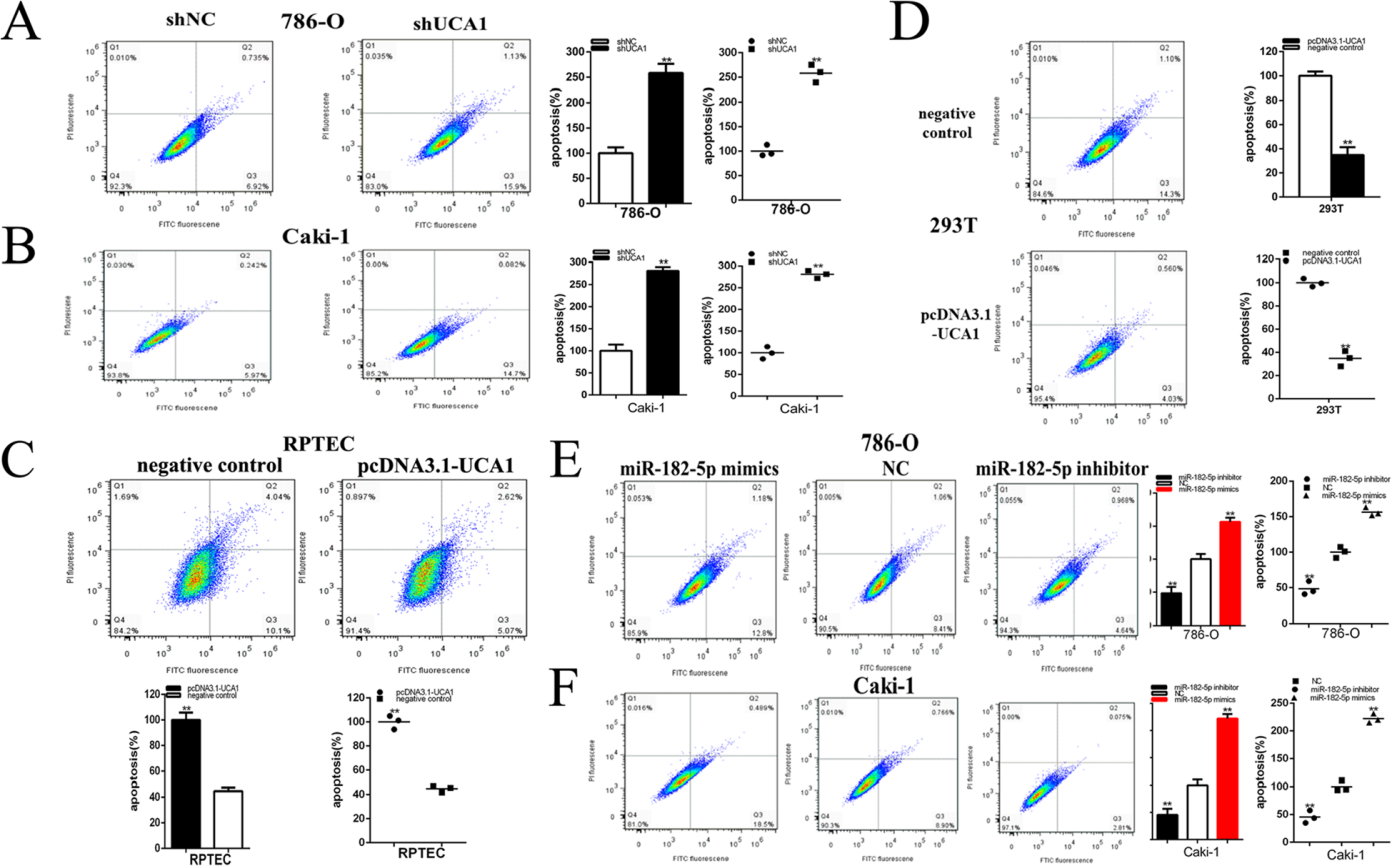
UCA1 and miR-182-5p impacted the renal cancer cell apoptosis**.** Apoptotic cells were measured after transfection of shRNA in the 786-O (**a**) and Caki-1(**b**) cell lines, of pcDNA3.1 in the RPTEC and 293 T cell line (**c** and **d**) and of miR-182-5p mimics or inhibitor in the 786-O (**e**) and Caki-1(**f**) cell lines by flow cytometry analysis .Assays were performed in triplicate,and data were shown as mean ± standard deviation (SD) of those biological replicates or samples (*P < 0.05, **P < 0.01)

Furthermore, we asked whether knockdown or overexpression of UCA1 and miR-182-5p can regulate cell apoptosis in renal cell lines after transfection of plasmids and miR-182-5p mimics or inhibitor and cell apoptosis was detected by flow cytometry assay. The apoptotic cells were increased remarkably in both cell lines after transfection with the shUCA1 as revealed by flow cytometry assay. Compared with control groups, the ratios of apoptosis were increased significantly by 2.58 times in 786-O (*P* < 0.001) and raised by2.81 times in Caki-1 (*P* < 0.001) (Fig. 5a and b) after transfected with the shUCA1.

Compared with control groups, the ratios of apoptosis were decreased significantly by55.20% in RPTEC (*P* < 0.001) (Fig. 5c) after transfected with the pcDNA3.1-UCA1.Compared with control groups, the ratios of apoptosis were decreased significantly by 65.22% in 293 T (*P* < 0.001) (Fig. 5d) after transfected with the pcDNA3.1-UCA1.

Compared with control groups, the ratios of apoptosis were increased significantly by1.57 times in 786-O (*P* < 0.001) and raised by 2.22 times in Caki-1 (*P* < 0.001) (Fig. 5e and f) after transfected with the miR-182-5p mimics. Compared with control groups, the ratios of apoptosis were decreased significantly by 51.68% in 786-O (*P* = 0.002) and reduced by 54.54% in Caki-1 (*P* = 0.003) (Fig. 5e and f) after transfected with the miR-182-5p inhibitor.

In a word, knockdown of UCA1 and up-regulation of miR-182-5p promoted cell apoptosis of renal cell lines and up-regulation of UCA1 and down-regulation of mi-182-5p suppressed cell apoptosis of renal cell lines.

### UCA1 was a target of miR-182-5p

**Fig. 6:**
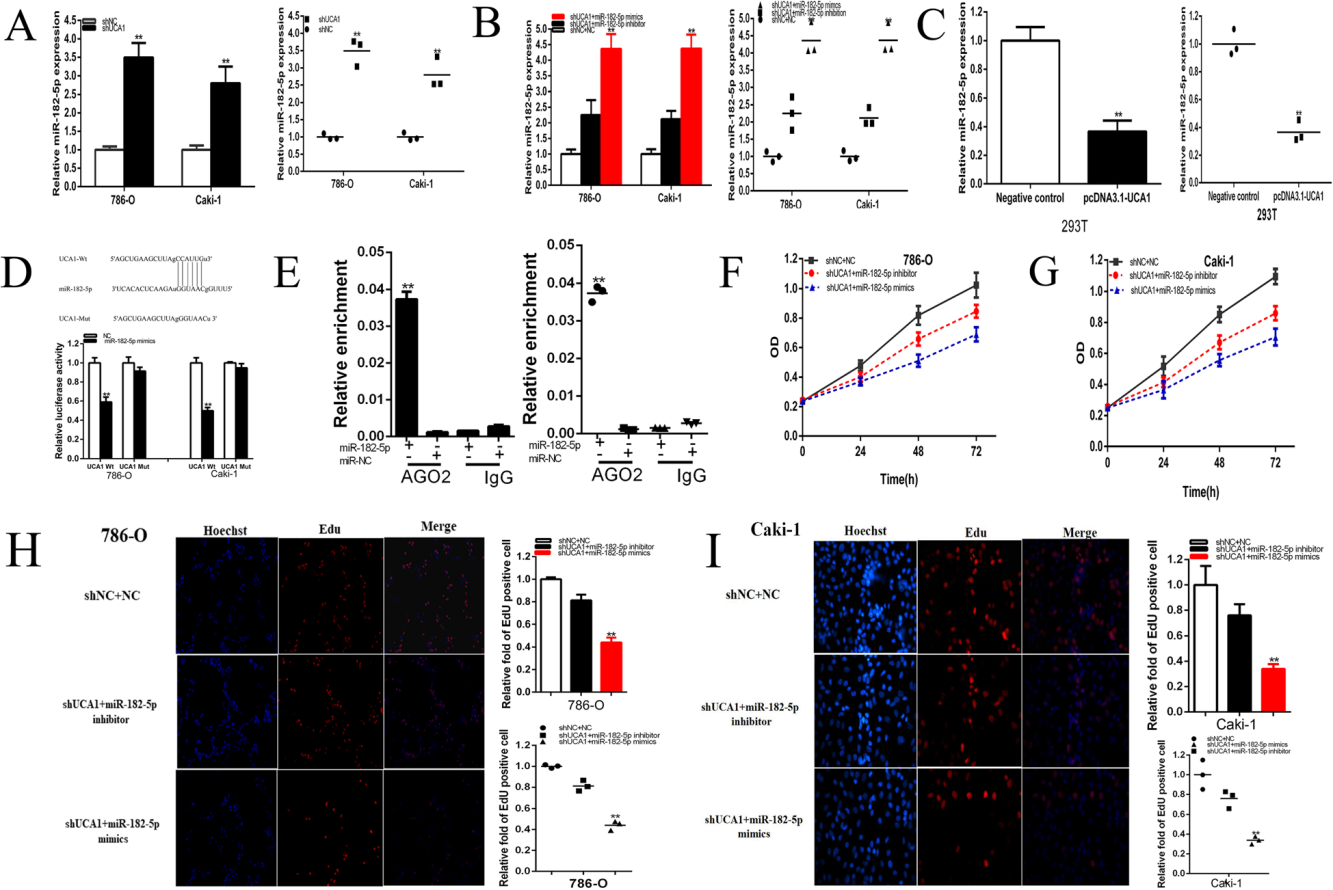
UCA1 was a target of miR-182-5p. The relative expression of miR-182-5p was up-regulated by shUCA1(**a**), and up-regulated by shUCA1 co-transfected miR-182-5p mimics (shUCA1 + miR-182-5p mimics)(**b**),down-regulated by pcDNA3.1-UCA1(**c**). Dual-luciferase reporter assays were performed in 786-o or Caki-1 cells co-transfected with UCA1-Wt or UCA1-Mut and miR-182-5p or NC (**d**). Anti-AGO2 RIP was performed in renal cells transfected with miR-182-5p mimics or NC, followed by RT-qPCR to detect UCA1 (**e**). ANOVA was used for the comparison of curves of cell proliferation. Cell proliferation was detected in both renal cancer cell lines after co-transfection with shNC+NC, shUCA1 + miR-182-5p inhibitor or shUCA1 + miR-182-5p mimics (**f** and **g**). Representative images of EdU assay were shown and the relative fold changes of EdU positive cells were detected after co-transfection with shNC+NC, shUCA1 + miR-182-5p inhibitor or shUCA1 + miR-182-5p mimics (**h** and **i**). Assays were performed in triplicate and data were shown as mean ± standard deviation (SD) of those biological replicates or samples (*P < 0.05, **P < 0.01)

Compared with shNC groups, the expressions of miR-182-5p were up-regulated by 3.494 times in 786-O (*P* < 0.001) and by 2.795 times in Caki-1 (*P* = 0.003) (Fig. 6a) in shUCA1 groups. Compared with shNC co-transfected NC groups, the expressions of miR-182-5p were up-regulated by 4.36 times in 786-O (*P* < 0.001) and by 4.37 times in Caki-1 (*P* < 0.001) (Fig. 6b) in shUCA1 co-transfected miR-182-5p mimics groups. Conversely, miR-182-5p inhibitor partially reversed the up-regulation expression effects of miR-182-5p induced by shUCA1, and decreased by 56.16% in 786-O and by 48.47% in Caki-1 cell lines. Compared with negative control groups, the expressions of miR-182-5p was down-regulated by 63.43%in 293 T (*P* < 0.001) in pcDNA3.1-CUCA1 groups (Fig. 6c).

Since lncRNAs can function as sponges or inhibitors of their interacting miRNAs, lncRNAs interacting with miRNAs were predicted. We wondered whether lncRNA exerted its effect through sponging miRNAs. The potential binding sites of UCA1 with miR-182-5p were predicted using RNAhybrid. Luciferase reporter assay was performed to confirm the predictions. We found that miR-182-5p mimics significantly inhibited luciferase activity of wild type reporter for lncRNA-UCA1, compared with the group of co-transfections with NC and pmirGLO-UCA1-Wt, the luciferase activity was decreased by 41.00% in 786–0(*P* < 0.001) and by 50.1% in Caki-1(*P* < 0.001) in the group of co-transfections with miR-182-5p mimics and pmirGLO-UCA1-Wt,however, miR-182-5p did not inhibit the luciferase activity of reporter vector containing the mutant binding sites of lncRNA-UCA1 (Fig. 6d). It has been known that miRNAs repress translation and degrade mRNA in an AGO2-dependent manner by binding to their targets. We conducted anti-AGO2 immunoprecipitation (RIP) in renal cells transiently overexpressing miR-182-5p to pull down the lncRNA-UCA1 using anti-AGO2 antibodies or control IgG, followed by RT-qPCR analysis for lncRNA-UCA1 levels. The results showed that lncRNA-UCA1 pulled down with anti-Ago2 antibodies was significantly enriched in cells transfected with miR-182-5p mimics compared to controls. (Fig. 6e), suggesting that miR-182-5p could directly target lncRNA-UCA1 in AGO2-dependent manner.

### The suppressive effects on renal cancer cells induced by UCA1 knockdown was mediated by miR-182-5p

**Fig. 7:**
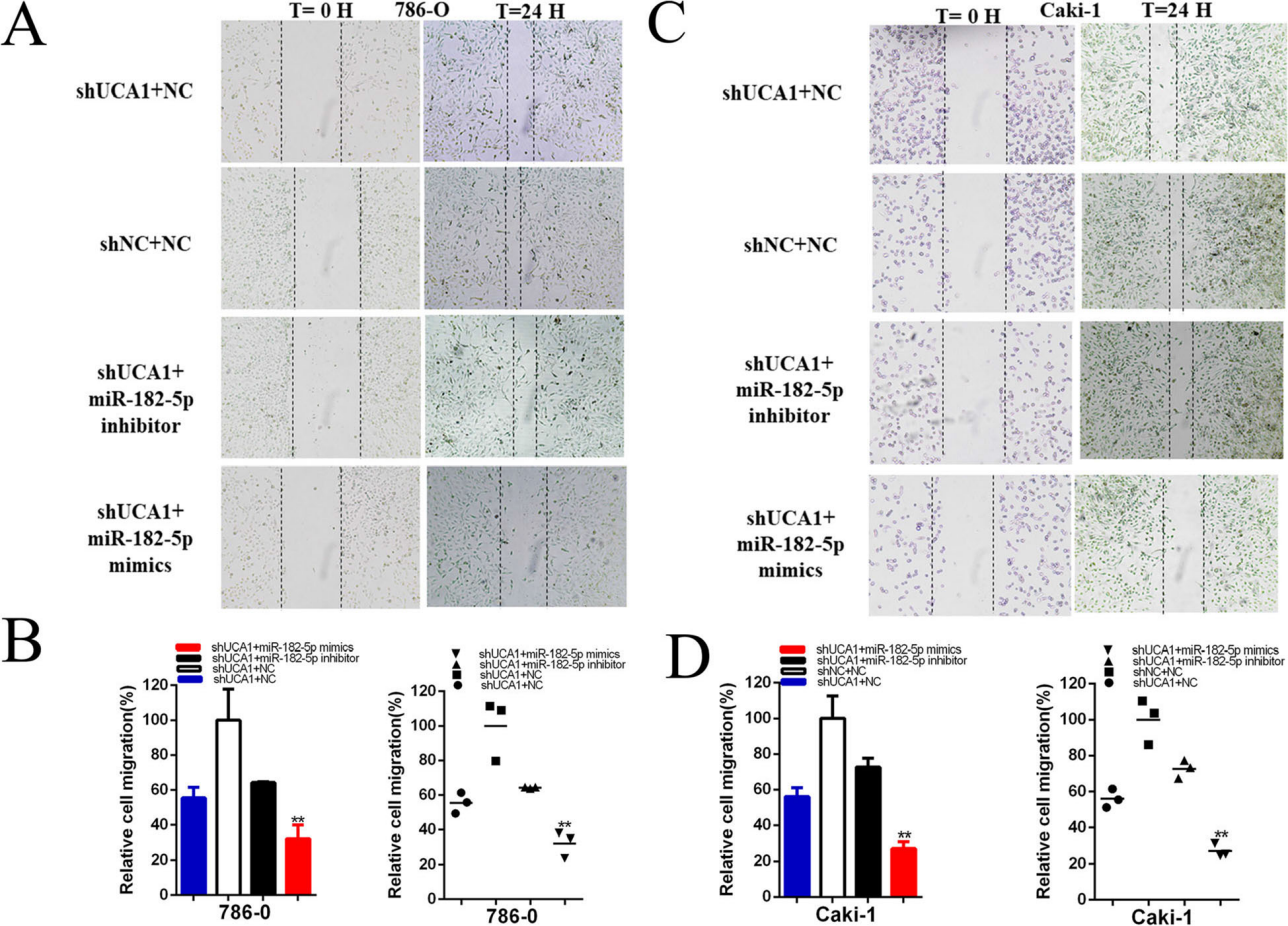
The effect of UCA1 and miR-182-5p after co-transfection on renal cancer cell migration. The relative cell migration after co-transfection with shNC+NC, shUCA1 + miR-182-5p inhibitor or shUCA1 + miR-182-5p mimics, and the representative images were as follow (**a**-**d**). Assays were performed in triplicate, and data were shown as mean ± standard deviation (SD) of those biological replicates or samples (*P < 0.05, **P < 0.01)

**Fig. 8:**
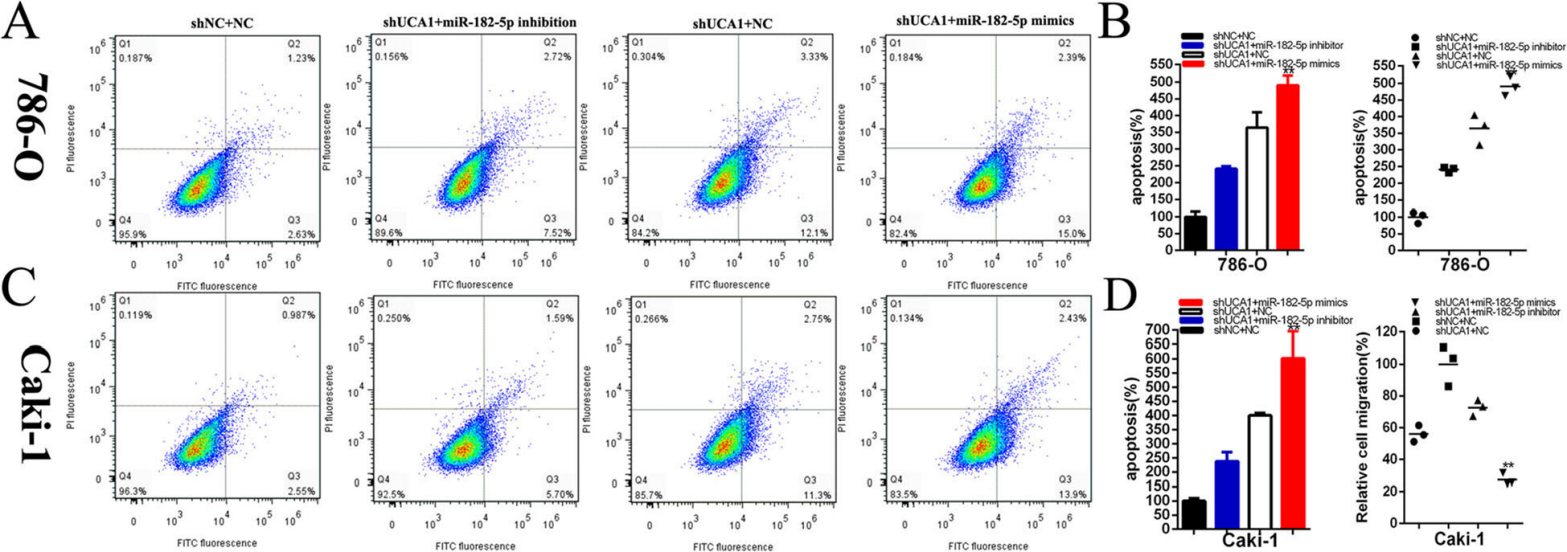
The effect of UCA1 and miR-182-5p after co-transfection on renal cancer cell apoptosis. The apoptotic cells were measured after co-transfection with shNC+NC, shUCA1 + miR-182-5p inhibitor or shUCA1 + miR-182-5p mimics by flow cytometry analysis (**a**-**d**). Assays were performed in triplicate, and data were shown as mean ± standard deviation (SD) of those biological replicates or samples (*P < 0.05, **P < 0.01)

After the luciferase reporter and RIP assay, UCA1 was confirmed to be a target of miR-182-5p. Whether miR-182-5p was involved in the inhibitory effects of shUCA1 renal cancer cells needed to be clarified. Cells that were stably transfected with shUCA1 were co-transfected with miR-182-5p mimics and showed stronger inhibitory effects on the proliferation (Fig. 6f-i), and migration(Fig. 7a-d) of renal cancer cells than the cells co-transfected with shNC co-transfected NC(shNC+NC), while apoptosis was significantly enhanced in shUCA1 co-transfected miR-182-5p(shUCA1 + miR-182-5p) group compared with shNC+NC group(Fig. 8a-d). Conversely, miR-182-5p inhibitor partially reversed the tumor suppressive effects induced by shUCA1**.**

The CCK-8 assay results demonstrated that shUCA1 co-transfected miR-182-5p(Fig. 6f and g) remarkably inhibited cell proliferation in both renal cancer cells (*p* < 0.01 in 786-O and *p* < 0.05 in Caki-1).Meantime, miR-182-5p inhibitor partially reversed the tumor suppressive effects induced by shUCA1 as what have been above (Fig. 6f and g) .

As shown in Fig. 6h and i, compared to shNC co-transfected NC (shNC+NC), EdU positive cells in shUCA1 co-transfected miR-182-5p mimics groups were significantly reduced by 55.89% in 786–0(*P* < 0.001) and by 65.67% in Caki-1(*P* = 0.001) (Fig. 6h and i). Conversely, miR-182-5p inhibitor partially reversed the EdU positive suppression induced by shUCA1, and increased by 37.12% in 786-O and by 42.59% in Caki-1 cell lines (Fig. 6h and i).

Compared to shNC+NC, the ratio of the relative migration in shUCA1 co-transfected miR-182-5p mimics groups were significantly reduced by 51.94% in 786–0(*P* = 0.002) and by 53.65% in Caki-1(*P* < 0.001). Correspondingly, miR-182-5p inhibitor partially reversed the ratio of the relative migration suppression induced by shUCA1, and increased by 20.62% in 786-O and by 18.63% in Caki-1 cell lines (Fig. 7a-d).

Compared to shNC+NC, the apoptosis in shUCA1 co-transfected miR-182-5p mimics groups were significantly elevated by 2.28 times in 786–0(*P* < 0.001) and by 2.47 times in Caki-1(*P* < 0.001). On the contrary, miR-182-5p inhibitor partially reversed the apoptosis accelerator induced by shUCA1, and decreased by 69.14% in 786-O and by 90.47% in Caki-1 cell lines (Fig. 8a-d).

### UCA1 positively regulates DLL4 expression via sponging miR-182-5p

**Fig. 9:**
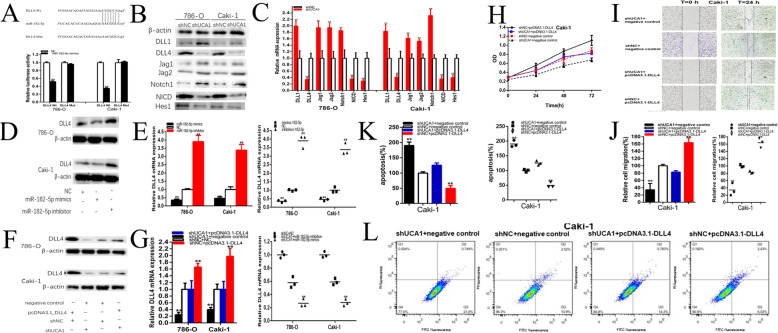
UCA1 positively regulates DLL4 expression via sponging miR-182-5p. The bioinformation analysis results showed that miR-182-5p and DLL4 have common putative binding sites, showed that the 3’UTR sequence of DLL4 is complementary to the seed sequence of miR-182-5p. Dual-luciferase reporter assay showed DLL4-Wt and miR-182-5p mimics co-transfection significantly inhibited luciferase activity (**a**). Knockdown of UCA1 decreased DLL4, NICD and Hes1 expression, and increased DLL1, Jag1, Jag2 and Notch1 expression in renal cancer cells (**b** and **c**). Overexpressing miR-182-5p decreased the expression of DLL4 and knockdown of miR-182-5p increased DLL4 expression in renal cancer cells (**d** and **e**). Overexpressing of DLL4 reversed malignant phenotypes inhibition of renal cancer cells induced by silencing shUCA1. The DLL4 specific vector (pcDNA3.1-DLL4) significantly reversed DLL4 expression inhibition induced by silencing UCA1 in renal cancer cells (**f** and **g**). Overexpressing DLL4 significantly reversed cell proliferation inhibition induced by silencing UCA1 (**h**). Overexpressing DLL4 significantly reversed cell migration inhibition induced by silencing UCA1 (**i** and **j**). Overexpressing DLL4 significantly reversed cell apoptosis promotion induced by silencing DLL4 (**k** and **l**). Assays were performed in triplicate, and data were shown as mean ± standard deviation (SD) of those biological replicates or samples (*P < 0.05, **P < 0.01)

The putative binding sites of DLL4 with miR-182-5p was predicted by the bioinformatics databases. While the binding sites were shown in Fig. 9a. Luciferase reporter assay was performed to verify the binding sites and binding effects. We found that miR-182-5p mimics significantly inhibited luciferase activity of wild type reporter for DLL4, compared with the group of co-transfections with NC and pmirGLO-UCA1-Wt, luciferase activity was decreased by 47.93% in 786–0 (*P* < 0.001) and by 64.83% in Caki-1 (*P* = 0.001) was observed in cells co-transfected of miR-182-5p and DLL4C-3’UTR-Wt,however, miR-182-5p did not inhibit the luciferase activity of reporter vector containing the mutant binding sites of DLL4 (Fig. 9a).

The results showed that UCA1 expression levels were statistically positively correlated with DLL4 expression levels in renal cancer cell lines and knockdown of UCA1 decreased DLL4 expression in renal cancer cells. Moreover, knockdown of DLL4 regulated Notch Signaling of renal cancer cells (Fig. 9b and c). The expression of Notch Signaling markers were determined using qRT-PCR, and western blotting. Knockdown of UCA1 increased DLL1, Jag1, Jag2 and Notch1expression and decreased DLL4, NICD and Hes1 expression in renal cancer cells.

We further determined whether UCA1 regulated the expression of DLL4 in renal cancer cells via miR-182-5p-dependent manner. We found overexpressing miR-182-5p decreased DLL4 expression in renal cancer cells (Fig. 9d and e). The results indicated that UCA1 positively regulates DLL4 expression via sponging miR-182-5p in renal cells.

### Overexpressing of DLL4 reverses malignant phenotypes inhibition of renal cancer cells induced by silencing UCA1

We further determined whether DLL4 regulated malignant phenotypes of renal cancer cells via DLL4- dependent manner. Our results showed that the DLL4 specific vector (pcDNA3.1-DLL4) significantly reversed the inhibition of DLL4 expression induced by silencing UCA1 in renal cancer cells (Fig. 9f and g). Meanwhile, we found DLL4 overexpression significantly reversed cell proliferation inhibition of renal cancer cells (Fig. 9h) induced by silencing UCA1. And DLL4 overexpression significantly reversed cell migration (Fig. 9i and j) of renal cancer cells induced by silencing UCA1. Moreover, DLL4 overexpression significantly reversed cell apoptosis promotion of renal cancer cells (Fig. 9k and l) induced by silencing UCA1. The results indicated that UCA1 promotes malignant phenotypes of renal cancer cells via DLL4-dependent manner.

### Knockdown of UCA1 inhibits tumorigenicity of renal cancer cells

**Fig. 10:**
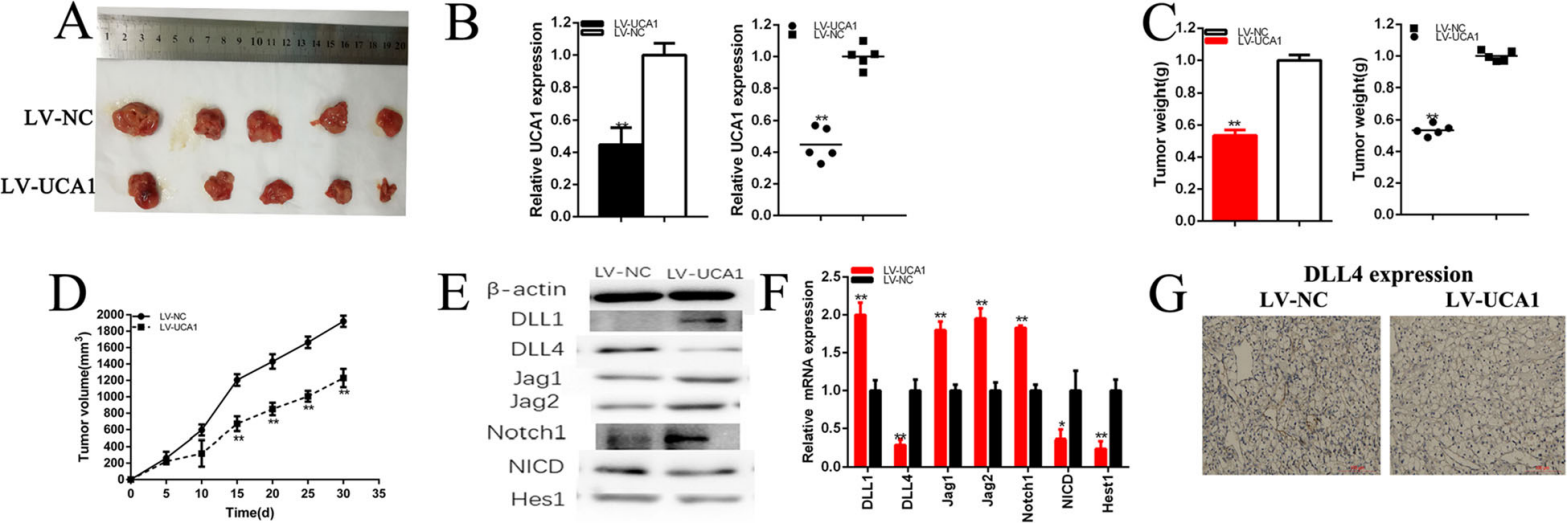
The effect of UCA1 on tumorigenicity of renal cancer cells. Tumors collected from mice were exhibited (**a**). The relative expression level of UCA1 was significantly down-regulated by LV-UCA1 (**b**). Tumor weight of LV-UCA1 or LV-NC treatment groups were measured and analyzed (**c**). Tumor volume curve of LV-UCA1 or LV-NC treatment groups were measured and analyzed (**d**). Knockdown of UCA1 decreased DLL4, NICD and Hes1 expression, and increased DLL1, Jag1, Jag2 and Notch1 expression in renal cancer cells (**e** and **f**). Knockdown of UCA1 decreased DLL4 expression of renal cancer cells in vivo (**g**). Data were shown as mean ± standard deviation (SD) of those biological replicates or samples (*P < 0.05, **P < 0.01)

**Fig. 11:**
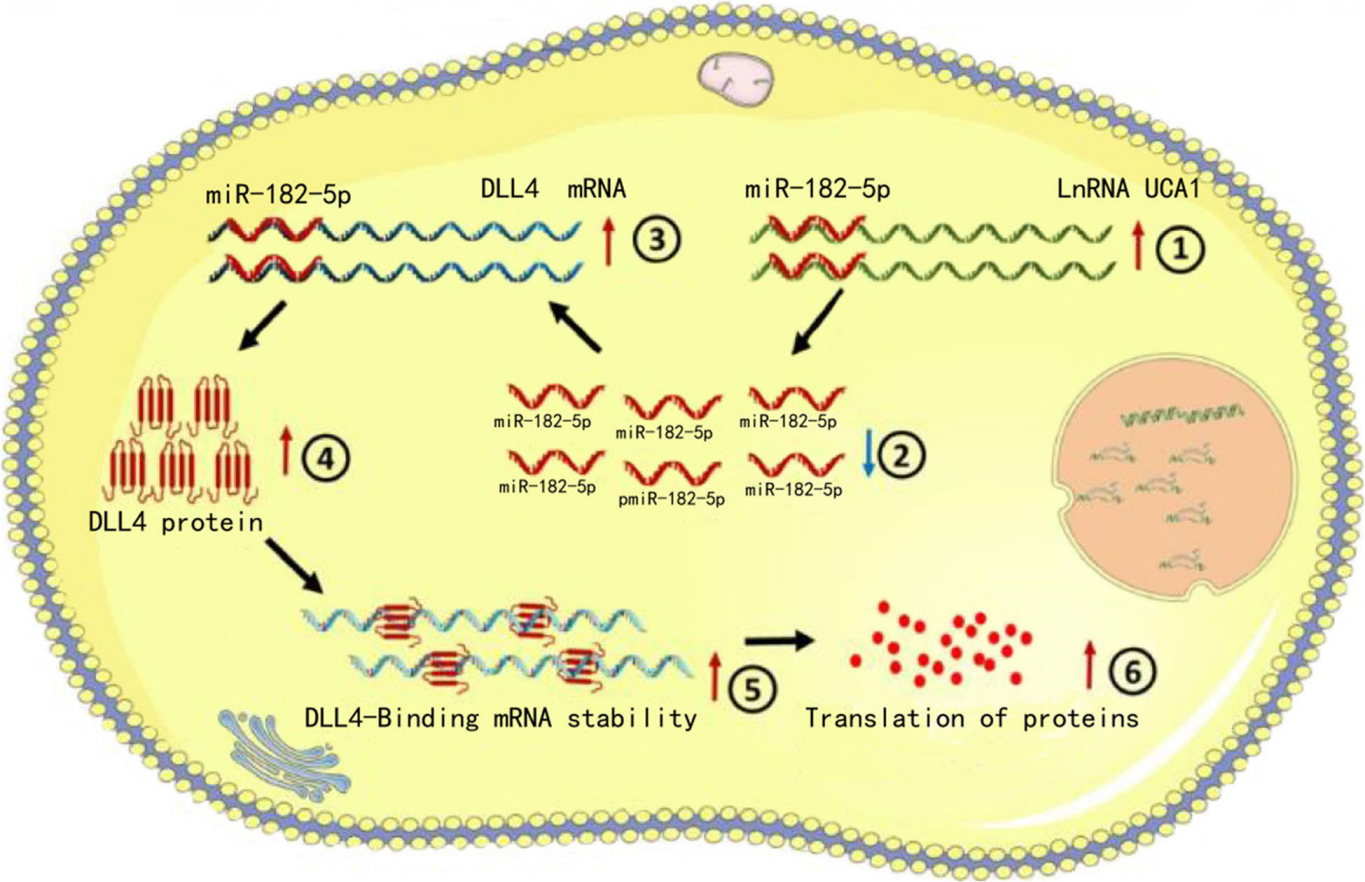
The schematic diagram of the oncogenic role of UCA1 in renal cancer cells. UCA1 functions as a miRNA sponge to positively regulate DLL4 expression through sponging miR-182-5p and subsequently promotes malignant phenotypes of renal cancer cells, thus playing an oncogenic role in renal cancer pathogenesis

We further determined whether UCA1 regulated tumorigenicity of renal cancer cells using generation of xenograft. We found knockdown of UCA1 inhibited the tumorigenicity of renal cancer cells in vivo (Fig. 10a-g). Tumors collected from mice were exhibited and measured (Fig. 10a). We found that decreased UCA1 expression was remarkably decreased compared to the LV-NC group of renal cancer cells in vivo (Fig. 10b). Tumor weight of LV-NC treatment group was greater than that in theLV-UCA1 group (Fig. 10c). Tumor growth of LV-NC treatment group was faster than that in the LV-UCA1 group (Fig. 10d). We found knockdown of UCA1 increased DLL1, Jag1, Jag2 and Notch1expression and decreased DLL4, NICD and Hes1 expression of renal cancer cells in vivo (Fig. 10e and f). Meanwhile, we found knockdown of UCA1 decreased DLL4 expression (Fig. 10g) of renal cancer cells in vivo. The results indicated that UCA1 promoted tumorigenicity of renal cancer cells via upregulating DLL4. As shown in Fig. 11, we found that UCA1 was significantly upregulated in renal cancer cells and UCA1 functioned as a miRNA sponge to positively regulate DLL4 expression through sponging miR-182-5p. Elevated DLL4 protein promoted transcription and translation of proteins operating in essential oncogenic signaling pathways, subsequently promoting malignant phenotypes of renal cancer cells.

## Discussion

The lncRNAs are important new members of non-coding RNA family, which are longer than 200 nucleotides. Accumulating evidences suggest that long non-coding RNAs (lncRNAs) perform important functions in regulation of genes which control tumor proliferation, apoptosis, and migration etc., and stretched our understanding of the biological behavior in diseases especially in carcinomas including renal cancer [2–10]. Furthermore, these previous studies indicate that lncRNAs are valuable biomarkers and therapeutic targets [11–15, 50]. The Long non-coding RNA UCA1 was located in chromosome 9p13.12, and has been found to be overexpressed in tumor tissues, such as esophageal squamous cell carcinoma,colorectal cancer, ovarian cancer, bile duct carcinoma and melanoma etc. [16–23].UCA1 are participated in the tumorigenesis and progression, functioning as an oncogene [24–30]. However, the relation between UCA1 and renal cancer is still unknown and mysterious particularly.

This is the first report to illustrate the function of UCA1 and miR-182-5p in renal cancer. In this research, we evaluated the expression patterns and clinical significances of UCA1 in renal cancer, and furthermore explored its possible function in renal cancer cells. Our study validated that UCA1 and miR-182-5p can be considered as a promising biomarker for the diagnosis of renal cancer. The expression of UCA1 was remarkably higher in renal cancer tissues and cell lines, and its up-regulation was positively correlated with differentiation and TNM stage in renal cancer. Furthermore, we explored the function of UCA1 in renal cancer cells and found that inhibition of UCA1 expression through UCA1 shRNA could suppress tumor cell proliferation, inhibited cell migration and induced apoptosis etc. To get a better insight to the role of UCA1, we over-expressed UCA1 in 293 T and PRTEC cell lines and it is found that overexpression of UCA1 could promote cell proliferation, migration and anti-apoptosis. In vivo studies demonstrated that UCA1 knockdown induced the smallest tumor volume and weight and so on in nude mice.

Accumulated evidences indicate the reciprocal inhibition between lncRNAs and miRNAs [16, 20, 22, 24, 25, 28–30], and our study is also a good example on the relation furthermore. LncRNAs function as miRNAs sponges or decoys that titrate the concentration of miRNAs and therefore prevent miRNAs binding specific mRNAs. The role of UCA1 as a miRNA sponge in neoplasms has been reported in several studies. UCA1 promotes the progression of melanoma by sponging miR-28-5p [22]. In Prostate cancer, UCA1 works as an oncogene by targeting miR-204 [28]. The above studies are consistent with our research. As lncRNAs could function as sponges, they also might be saturated with miRNAs. Therefore, distinct expressions of lncRNAs and miRNAs in different tissues may induce different binding actions. Bioinformatics prediction was performed in order to investigate the underlying mechanism of UCA1 in renal cancer in our study, and miR-182-5p were predicted as potential targets. The prediction was confirmed by luciferase assay, and meanwhile miR-182-5p was found to be up-regulated in UCA1 knockdown cell lines. Furthermore, miR-182-5p were found to be down-regulated in renal cancer specimens and cell lines. And its down-regulation was positively correlated with differentiation and TNM stage in renal cancer. Over-expression of miR-182-5p respectively inhibited the malignant behaviors of renal cancer cells, and inhibited cell proliferation, migration and promoted apoptotic while the opposite effects were found in renal cancer cells with down-regulated miR-182-5p. MiR-182-5p plays tumor-suppression and anti-angiogenesis roles through altering the microenvironment and regulating DLL4 expression. Furthermore, we found that UCA1 knockdown combined with miR-182-5p over-expression significantly suppressed the malignant behaviors of renal cancer cells. Down-regulation of miR-182-5p could partially rescue the inhibition induced by UCA1 knockdown. This suggested that the suppression of malignancy caused by UCA1 knockdown might result from up-regulating of miR-182-5p.

As we known, miRNAs regulate lives’ activities by means of the protein indirect role and regulate target genes by binding to the 3’UTR of specific mRNAs and cause degradation or transcriptional inhibition of target genes [31–36]. Biological softwares were used to predict the possible target genes of miR-182-5p to investigate the mechanisms of these miRNAs in renal cancer. DLL4 was a shared target gene of miR-182-5p, which was identified by luciferase reporter assay. Overexpression of miR-182-5p suppressed DLL4 protein expression, while down-regulation of miR-182-5p enhanced DLL4 expression. Thus, the expression of DLL4 was negatively correlated with the expression of miR-182-5p. Moreover, DLL4 had been proved to be involved in the malignant behaviors of renal cancer in previous studies [37].

Delta-like ligand 4 (DLL4), one of the ligands of Notch receptors, is predominantly expressed in the endothelial cells and has been shown to play a pivotal role in regulating tumor angiogenesis [38–45]. Some studies reveal a role for DLL4 in tumorigenesis in several cancers, including T acute lymphoblastic leukemia (T-ALL) [46], and glioblastoma [47] etc. We found that overexpressing of DLL4 reverses malignant phenotypes inhibition of renal cancer cells induced by silencing UCA1. DLL4 overexpression significantly reversed cell proliferation inhibition and migration of renal cancer cells induced by silencing UCA1, and significantly reversed cell apoptosis promotion of renal cancer cells induced by silencing UCA1. DLL4-NOTCH signaling is critical for the progression of renal cancer cells.

In brief, further experiments demonstrated that knockdown of UCA1 increased miR-182-5p expression and subsequently inhibited the expression of DLL4 in a ceRNA-dependent manner. Moreover, knock-down of miR-182-5p reversed DLL4 expression and DLL4 overexpression reversed the malignant phenotype inhibition of renal cancer cells induced by silencingUCA1.This study is giving us a novel point of view for specific molecular targets in human cancers, especially renal cancers, and it deepened our understanding of the relationship between lncRNA,miRNA and proteins in disease progression, and provides us a direction to further discover the diseases’ occurrence mechanism in the future, and to overcome and conquer the fatal disease etc. Maybe, it can be converted into the more freshly and validly curative mean for the renal carcinomas. It may be a bright development prospect to radically cure renal cancer that could alter the current dilemma of treatment for the advanced renal cancer.

## Conclusions

Our study reveals that UCA1 functions as a miRNA sponge to positively regulate DLL4 expression through sponging miR-182-5p and subsequently promotes the malignant phenotypes of renal cancer cells, thus playing an oncogenic role in renal cancer pathogenesis. The results of this study provide a new basis for studying the mechanism of the occurrence and development of renal cancer. Cumulatively, our results suggest that UCA1-miR-182-5p-DLL4 axis is involved in proliferation migration, apoptosis and progression of renal cancer. UCA1 is a powerful tumor biomarker, which highlight its potential clinical utility as a promising diagnostic and therapeutic target of renal cancer.

## Supplementary information

**Additional file 1: Table S1.** The primers for real-time QPCR. **Table S2.** Antibodies used for Western blots.

## Data Availability

All authors are responsible for the truth and reliability of the article data.

## Abbreviations

ANOVA: Analysis of Variance
CCK-8: Cell Counting Kit-8
CeRNA: Competing endogenous RNA
DLL4: Delta-like ligand 4
EdU: Ethynyl-2-deoxyuridine
IDVs: Integrated density values
lncRNAs: Long non-coding RNAs
LV-RNA: Lentivirus shRNA
miRNAs: MicroRNAs
PVDF: Polyvinylidene Fluoride
qPCR: Quantitative polymerase chain reaction
RCC: Renal cell carcinoma
SDS-PAGE: SDS-polyacrylamide gel electrophoresis
shNC: Short hairpin negative control
shRNAs: Short hairpin RNAs
T-ALL: T acute lymphoblastic leukemia
TNM: Tumor Node Metastasis
UCA1: Urothelial cancer associated 1
