# Correction to: Long non-coding RNA UCA1 promotes malignant phenotypes of renal cancer cells by modulating the miR-182-5p/DLL4 axis as a ceRNA

**DOI:** 10.1186/s12943-021-01433-4

**Published:** 2022-01-03

**Authors:** Wei Wang, Wentao Hu, Ya Wang, Yong An, Lei Song, Panfeng Shang, Zhongjin Yue

**Affiliations:** 1 Department of Urology, Institute of Urology, Gansu Nephro-Urological Clinical Center, Key Laboratory of Urological Diseases in Gansu Province, Lanzhou University Second Hospital, Lanzhou, 730030 Gansu China; 2 School of Radiation Medicine and Protection, Medical College of Soochow University, Collaborative Innovation Center of Radiological Medicine of Jiangsu Higher Education Institutions, Suzhou, 215123 China; 3 Department of Nephrology, Second Hospital Lanzhou University Second Hospital, Lanzhou, 730000 Gansu China; 4 Medical School, Northwest Min Zu University, Lanzhou, 730030 Gansu China

**Correction to: Mol Cancer 19, 18 (2020)**

**https://doi.org/10.1186/s12943-020-1132-x**

Following publication of the original article [1], minor errors were identified in the images presented in Figs. 3, 4, and 6; specifically:

·Fig. 3d – miR-182-5p inhibitor images for Hoechst, Edu and Merge (bottom row)
·Fig. 4a – shUCA1 at 0 h (bottom left panel)
·Fig. 6i – shNC+NC images for Hoechst, Edu and Merge (top row)

The authors provided the journal with the original data files. The corrected figures are provided here. The correction does not have any effect on the results or conclusions of the paper. The original article has been corrected.

**Fig. 3:**
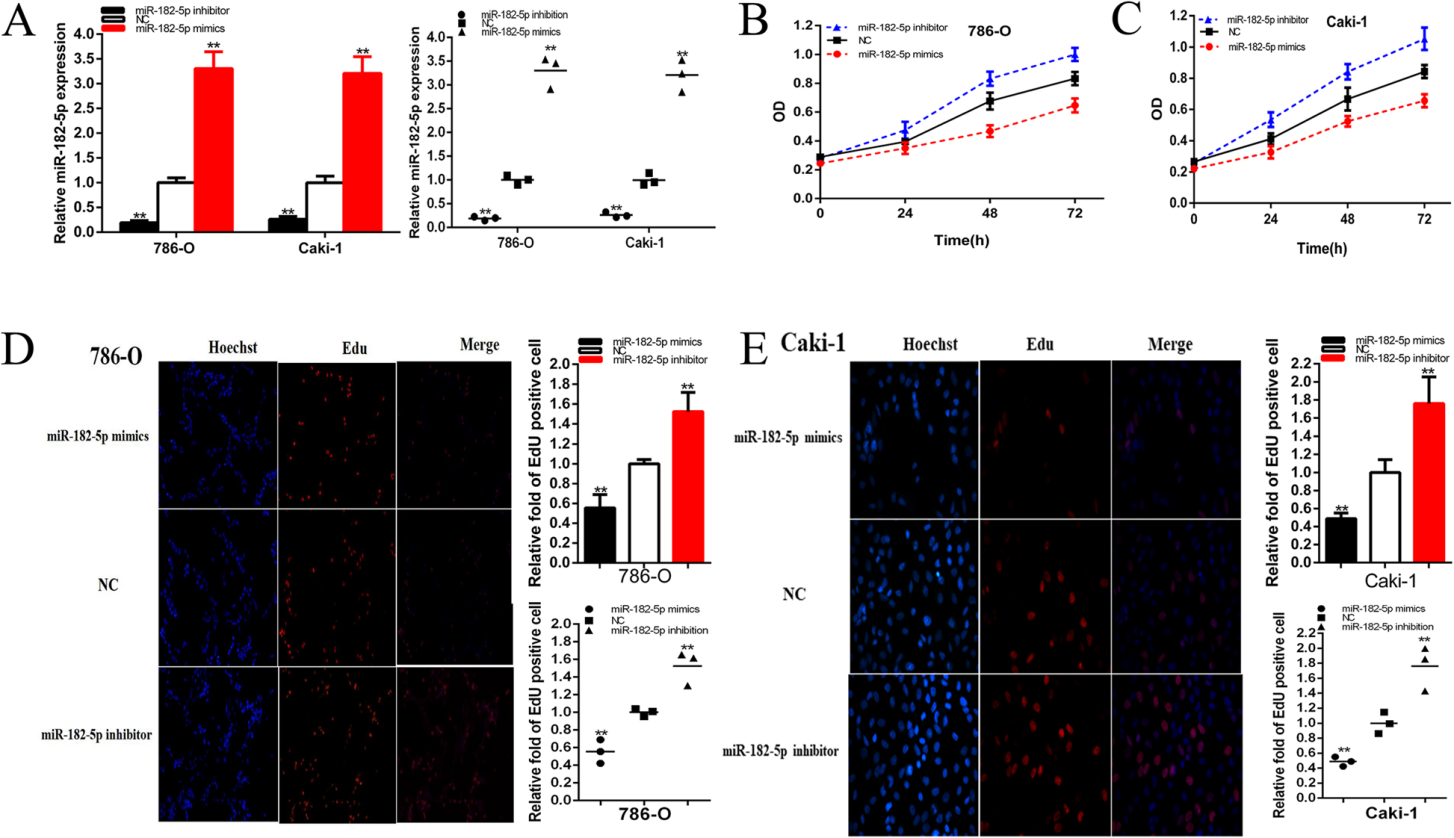
Knockdown and overexpression of miR-182-5p promote or inhibited cell proliferation. The relative expression level of miR-182-5p was significantly down-regulated by miR-182-5p inhibitor and up-regulated by miR-182-5p mimics (**a**). ANOVA was used for the comparison of curves of cell proliferation. Cell proliferation was detected in both renal cancer cells after transfection of miR-182-5p inhibitor and miR-182-5p mimics (**b** and **c**). Representative images of EdU assay and the relative fold changes of EdU positive cells were detected by miR-182-5p inhibitor and miR-182-5p mimics (**d** and **e**). Assays were performed in triplicate, and data were shown as mean ± standard deviation (SD) of those biological replicates or samples (*P < 0.05, **P < 0.01)

**Fig. 4:**
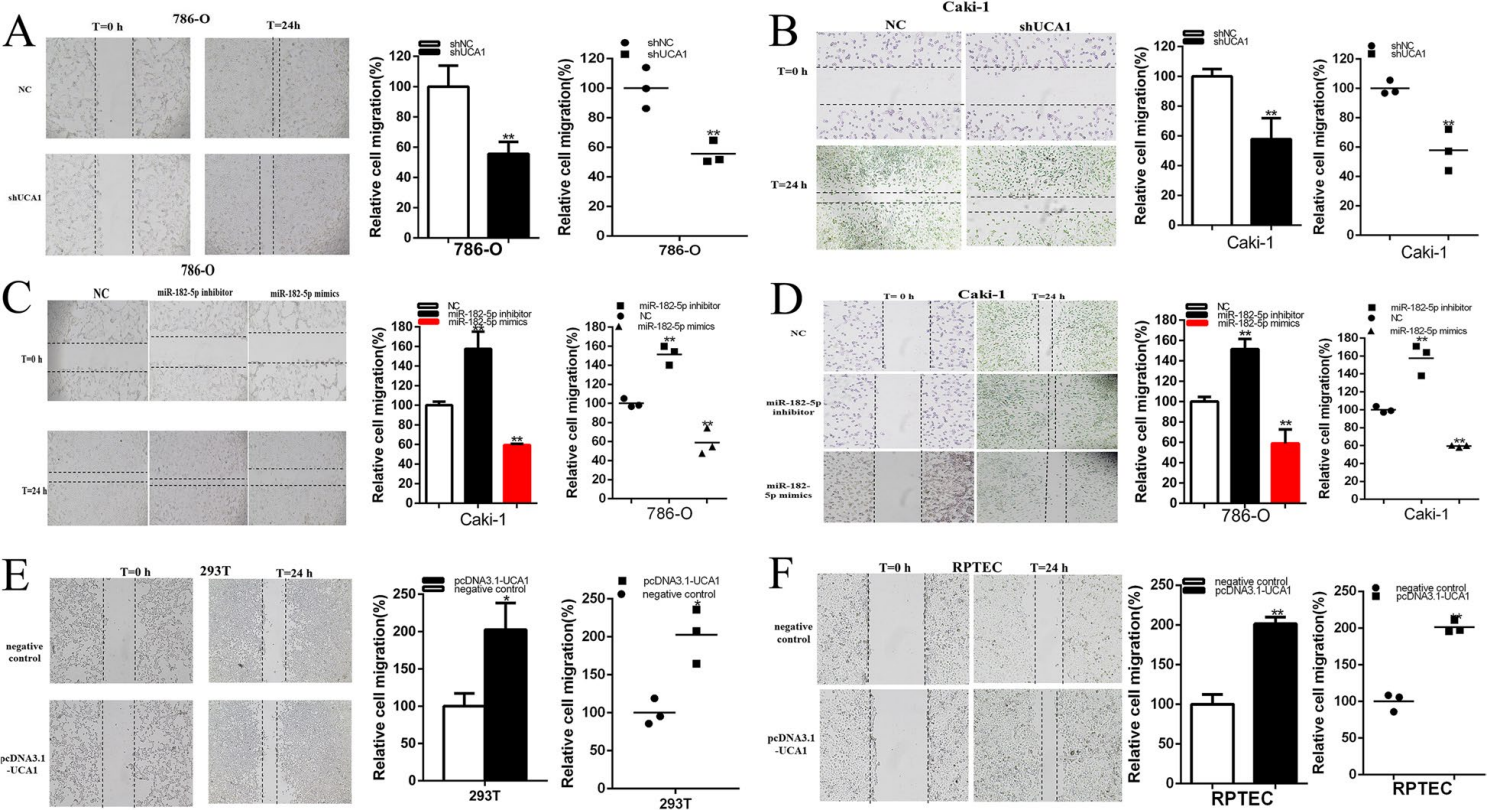
UCA1 and miR-182-5p impacted the renal cancer cell migration. The relative cell migration was suppressed after transfection of shRNA in the 786-O and Caki-1(**a** and **b**) cell lines. The relative cell migration was suppressed or promoted after transfection of miR-182-5p mimics or inhibitor in the 786-O and Caki-1(**c** and **d**) cell lines. The relative cell migration was promoted after transfection of pcDNA3.1-UCA1 in the 293 T and RPTEC cell lines (**e** and **f**). Assays were performed in triplicate, and data were shown as mean ± standard deviation (SD) of those biological replicates or samples (*P < 0.05, **P < 0.01)

**Fig. 6:**
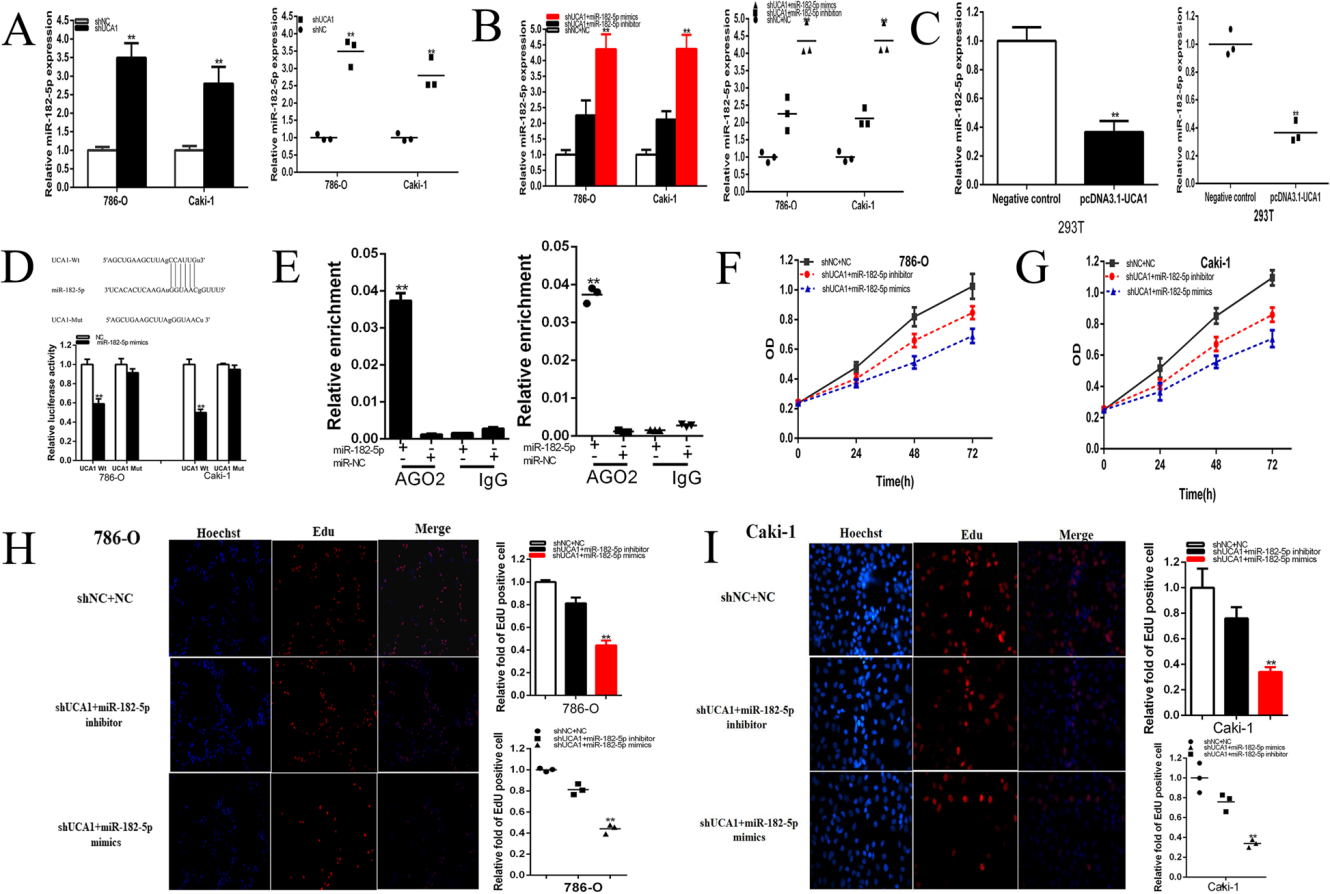
UCA1 was a target of miR-182-5p. The relative expression of miR-182-5p was up-regulated by shUCA1(**a**), and up-regulated by shUCA1 co-transfected miR-182-5p mimics (shUCA1 + miR-182-5p mimics)(**b**), down-regulated by pcDNA3.1-UCA1(**c**). Dual-luciferase reporter assays were performed in 786-o or Caki-1 cells co-transfected with UCA1-Wt or UCA1-Mut and miR-182-5p or NC (**d**). Anti-AGO2 RIP was performed in renal cells transfected with miR-182-5p mimics or NC, followed by RT-qPCR to detect UCA1 (**e**). ANOVA was used for the comparison of curves of cell proliferation. Cell proliferation was detected in both renal cancer cell lines after co-transfection with shNC+NC, shUCA1 + miR-182-5p inhibitor or shUCA1 + miR-182-5p mimics (**f** and **g**). Representative images of EdU assay were shown and the relative fold changes of EdU positive cells were detected after co-transfection with shNC+NC, shUCA1 + miR-182-5p inhibitor or shUCA1 + miR-182-5p mimics (**h** and **i**). Assays were performed in triplicate and data were shown as mean ± standard deviation (SD) of those biological replicates or samples (*P < 0.05, **P < 0.01)
