# Supplementary material for: Long non-coding RNA UCA1 promotes malignant phenotypes of renal cancer cells by modulating the miR-182-5p/DLL4 axis as a ceRNA
Source: Mol Cancer. 2020 Jan 29;19:18. doi: 10.1186/s12943-020-1132-x (PMC6988374; doi:10.1186/s12943-020-1132-x)
Supplement: Supplementary file 1 — Additional file 1: Table S1. The primers for real-time QPCR. Table S2. Antibodies used for Western blots. [file 12943_2020_1132_MOESM1_ESM.docx]

**Additional file 1**

Table S1. The primers for real-time QPCR

| Gene | Forward or Reverse | Primer sequence |
| --- | --- | --- |

| Dll1 | Forward | 5’-GGA CTG AAA GCC AGA CGA AG-3’ |
| --- | --- | --- |
|  | Reverse | 5’-TCT TCA AAG ACC CAG GGA TG-3’ |
| Dll4 | Forward | 5’-CCT CTC GAA CTT GGA CTT GC-3’ |
|  | Reverse | 5’-AGC TCC TGC TTA ATG CCA AA-3’ |
| Jag1 | Forward | 5’-CAG TGC CTC TGT GAG ACC AA-3’ |
|  | Reverse | 5’-AGG GGT CAG AGA GAC AAG CA-3’ |
| Jag2 | Forward | 5’-CAG ATC CGA TGA CGC TGT GA-3’ |
|  | Reverse | 5’-GGC TTC TTT GCA TTC TTT GC-3’ |
| Notch1 | Forward | 5’-TGA GAC TGC CAA AGT GTT GC-3’ |
|  | Reverse | 5’-GTG GGA GAC AGA GTG GGT GT-3’ |
| Hes1 | Forward | 5’- GGA GAG GCT GCC AAG GTT TT-3’ |
|  | Reverse | 5’- GCA AAT TGG CCG TCA GGA-3’ |
| miR182-5p | Forward | 5’-TTAGGAACCCTCCTCTCTC-3’ |
|  | Reverse | 5’- ACT TTC GTT CTT GAG GAA TG-3’ |
| UCA1 | Forward  Reverse | 5'-CTCTCCATTGGGTTCACCATTC-3'  5’-CGGTGATGTGAAGAAGGA-3’ |
| U6 snRNA  GAPDH. | Forward  Forward  Reverse | 5’-GCTTCGGCAGCACATATACTAAAAT-3’  5’-CGCTCTCTGCTCCTCCTGTTC-3’  5’ATCCGTTGACTCCGACCTTCAC-3’ |
| Dll4-3’-UTR-wnt | Forward | 5’-GCG GGC TCG AGT CCT AGA GAG GTC CAG AGC ACC-3’ |
|  | Reverse | 5’-AAT GCG GCC GCA CAT GGC AAA CCC AAG CAT AC-3’ |
| Dll4-3’-UTR-mut | Forward | 5’-TTC TGC ATA CAA ATG ATT GCA TCC TGT ATG GGA-3’ |
|  | Reverse | 5’-GAT GCA ATC ATT TGT ATG CAG AAG GAA GGC CCA-3’ |

Note: Dll1, delta-like 1; Dll4, delta-like4; Jag1, jagged 1; Jag2, jagged 2; Notch1, Notch1; Hes1, hairy and enhancer of split 1;

Table S2. Antibodies used for Western blots

| Antibody | Description | Dilution | Supplier | City | Country |
| --- | --- | --- | --- | --- | --- |
| Anti-Dll1 | Rabbit polyclonal | 1:1000 | Abcam | Cambridge | USA |
| Anti-Dll4 | Rabbit polyclonal | 1:1000 | Abcam | Cambridge | USA |
| Anti Notch1 | Rabbit polyclonal | 1:1000 | Abcam | Cambridge | USA |
| Anti-NICD | Rabbit polyclonal | 1:800 | Abcam | Cambridge | USA |
| Anti-Hes1 | Rabbit monoclonal | 1:1500 | Abcam | Cambridge | USA |
| Anti-Jag1 | Rabbit polyclonal | 1: 500 | Santa Cruz | Santa Cruz | USA |
| Anti-Jag2 | Rabbit polyclonal | 1:1000 | Santa Cruz | Santa Cruz | USA |
| Anti-β-actin  AGO2  IgG | Mouse monoclonal  Mouse  Monoclonal  Mouse  Monoclonal | 1:1000  1:1000  1:1000 | Beyotime  Santa Cruz  Santa Cruz | Shanghai  Santa Cruz  Santa Cruz | China  USA  USA |

**Table S3:** Correlation between UCA1 expression and clinicopathological characteristics of renal cell cancer patients^[1,2,3]^（Papillary renal carcinoma）

Characteristics Total Expression of UCA1 P value

High Low

(n=20) (n=10)

Gender

Male 17 13(48.1%) 14(51.9%) 0.254

Female 13 7(30.4%) 6(69.6%)

Tumor size (cm)

≤7cm 22 14(63.6%) 8(36.4%) 0.682

＞7cm 8 6(75.0%) 2(25.0%)

Age

$\leq$ 55 12 7(58.3%) 5(41.7%) 0.461

|  |
| --- |

>55 18 13(72.2%) 5(27.8%)

Differentiation

Moderate/poor 21 17(81.0%) 4(19.0%) 0.030*

Well 9 3(33.3%) 6((66.7%)

TNM stage)

T0-1 20 19(95.0%) 1(5.00%) <0.001**

T2 or above 10 1(10.0%) 9(90.0%)

Lymph node metastasis(N)

N0 25 16(64.0%) 9(36.0%) 0.640

N1 or above 5 48(0.0%) 1(20.0%)

(*P < 0.05, **P < 0.01)

TNM according to staging TNM of American Joint Committee on Cancer (AJCC) in 2010.

[1]. Zhuang C, Ma Q, Zhuang C, et al. LncRNA GClnc1 promotes proliferation and invasion of bladder cancer through activation of MYC[J]. FASEB journal: official publication of the Federation of American Societies for Experimental Biology,2019,33(10): 11045-11059.DOI:10.1096/fj.201900078

[2]. Chen Zhuang C, Liu Y, et al. Tetracycline-inducible shRNA targeting antisense long non-coding RNA HIF1A-AS2 represses the malignant phenotypes of bladder cancer[J]. Cancer letters,2016,376(1): 155-164.DOI: 10.1016/j.canlet.2016.03.037

[3]. Gong S, Qu X, Yang S, et al. RFC3 induces epithelial‑mesenchymal transition in lung adenocarcinoma cells through the Wnt/β‑catenin pathway and possesses prognostic value in lung adenocarcinoma[J]. International journal of molecular medicine,2019,44(6): 2276-2288.DOI:10.3892/ijmm.2019.4386

**Table S4** Correlation between DLL4 expression and clinicopathological characteristics of renal cell cancer patients^[1,2,3]^（Papillary renal carcinoma）

Characteristics Total Expression of DLL4 P value

High Low

(n=22) (n=8)

Gender

Male 17 14(82.4%) 3(17.6%) 0.242

Female 13 8(61.5%) 5(38.5%)

Tumor size (cm)

≤7cm 22 17(77.3%) 5(22.7%) 0.643

＞7cm 8 5(62.5%) 3(27.5%)

Age

$\leq$ 55 12 7(58.3%) 5(41.7%) 0.210

|  |
| --- |

>55 18 15(83.3%) 3(16.7%)

Differentiation

Moderate/poor 21 18(85.7%) 3(14.3%) 0.032*

Well 9 4(44.4%) 5((55.6%)

TNM stage)

T0-1 20 18(90.0%) 2(10.0%) 0.007**

T2 or above 10 4(40.0%) 6(60.0%)

Lymph node metastasis(N)

N0 25 19(76.0%) 6(24.0%) 0.589

N1 or above 5 3(60.0%) 2(40.0%)

(*P < 0.05, **P < 0.01)

TNM according to staging TNM of American Joint Committee on Cancer (AJCC) in 2010.

[1]. Zhuang C, Ma Q, Zhuang C, et al. LncRNA GClnc1 promotes proliferation and invasion of bladder cancer through activation of MYC[J]. FASEB journal: official publication of the Federation of American Societies for Experimental Biology,2019,33(10): 11045-11059.DOI:10.1096/fj.201900078

[2]. Chen Zhuang C, Liu Y, et al. Tetracycline-inducible shRNA targeting antisense long non-coding RNA HIF1A-AS2 represses the malignant phenotypes of bladder cancer[J]. Cancer letters,2016,376(1): 155-164.DOI: 10.1016/j.canlet.2016.03.037

[3]. Gong S, Qu X, Yang S, et al. RFC3 induces epithelial‑mesenchymal transition in lung adenocarcinoma cells through the Wnt/β‑catenin pathway and possesses prognostic value in lung adenocarcinoma[J]. International journal of molecular medicine,2019,44(6): 2276-2288.DOI:10.3892/ijmm.2019.4386
